# FAS gene expression, prognostic significance and molecular interactions in lung cancer

**DOI:** 10.3389/fonc.2024.1473515

**Published:** 2024-10-02

**Authors:** Kaimin Li, Shing Cheng Tan, Zhihao Yang, Chenwei Li

**Affiliations:** ^1^ Department of Thoracic Surgery, The First Affiliated Hospital of Ningbo University, Ningbo, Zhejiang, China; ^2^ UKM Medical Molecular Biology Institute, Universiti Kebangsaan Malaysia, Kuala Lumpur, Malaysia

**Keywords:** apoptosis, bioinformatics, data mining, FAS, *in silico*, lung carcinoma

## Abstract

**Introduction:**

FAS has been implicated in the development of various cancers, but its involvement in lung cancer has not been systematically characterized. In this study, we performed data mining in online tumor databases to investigate the expression, methylation, alterations, protein interactions, co-expression and prognostic significance of FAS in lung cancer.

**Method:**

The expression, prognostic significance and molecular interactions of FAS in lung cancer was mined and analyzed using GENT2, GEPIA2, UALCAN, cBioPortal, STRING, GeneMANIA, UCSC Xena, Enrichr, and OSluca databases. FAS expression was subsequently investigated at the protein level in samples from 578 lung cancer patients to understand its protein-level expression. *In vitro* validation of FAS gene expression was performed on H1299, H1993, A549 and HBE cell lines.

**Result:**

We found that the expression of FAS was significantly downregulated in both lung adenocarcinoma (LUAD) and lung squamous cell carcinoma (LUSC) compared to normal lung tissue. In addition, we observed a higher level of FAS promoter methylation in LUSC tissue than in normal tissue. FAS alterations were rare (1.9%) in lung cancer samples, with deep deletions being more common than missense mutations, which occurred mainly in the TNFR-like cysteine-rich domain and the death domain. We also identified a list of proteins interacting with FAS and genes co-expressed with FAS, with LUAD having 11 co-expressed genes and LUSC having 90 co-expressed genes. Our results also showed that FAS expression has limited prognostic significance (HR=1.302, 95% CI=0.935-1.139, P=0.530). Protein level investigation revealed that FAS expression varied among individuals, with nTPM values ranging from 5.2 to 67.2.

**Conclusion:**

This study provides valuable insights into the involvements and characteristics of FAS in lung cancer. Further studies are needed to investigate the clinical significance of FAS alterations in lung cancer and to explore the potential of targeting FAS for therapeutic intervention.

## 1 Introduction

Lung cancer is the leading cause of cancer deaths worldwide. In 2020 alone, lung cancer was responsible for about 2.2 million new cases and nearly 1.8 million deaths (1). The cancer is more common in men than in women, accounting for 14.3% and 11.4% of all new cancer cases, respectively. Lung cancer also has a low 5-year survival rate of about 10-20%, due in part to the fact that the disease is often detected at an advanced stage, and accounts for nearly one-fifth of all cancer deaths (1, 2). The number of new lung cancer cases and deaths is expected to continue to increase over the next 15 years, continuing the upward trend in lung cancer incidence and mortality (3). An aging population and continued tobacco use in many regions of the world are predicted to be the main causes of this increase (4).

There are two main histologic types of lung cancer, namely small cell lung cancer (SCLC) and non-small cell lung cancer (NSCLC) (5). SCLC is a rare but aggressive form of lung cancer. On the other hand, NSCLC is the most common form of lung cancer, accounting for about 80-85% of all cases (5). NSCLC includes more subtypes than SCLC, including lung adenocarcinoma (LUAD) and squamous cell carcinoma (LUSC), which arises in glandular and squamous cells, respectively. However, the role of FAS in lung cancer subtypes, particularly LUAD and LUSC, remains poorly understood concerning its gene expression, mutational landscape, and clinical relevance.

Like other cancers, lung cancer exhibits several hallmarks that are commonly associated with the disease (6, 7). One of these hallmarks is the evasion of apoptosis, a mechanism in which the Fas receptor plays an important role. The transmembrane receptor belongs to the TNF receptor (TNFR) superfamily, consists of 319 amino acids and has a size of about 48 kDa. The protein consists of a TNFR-like domain at its N-terminus, which is rich in cysteines and necessary for interaction with Fas ligand (FasL) (8). It also contains a death domain near the carboxyl terminus that is essential for interaction with FasL. When FasL binds to Fas receptor homotrimers, the receptor is activated and recruits the adaptor protein, Fas-associated death domain (FADD), which in turn recruits procaspase-8 to form the death-inducing signaling complex (DISC) (9). Procaspase-8 is cleaved in the DISC into the active caspase-8, which then triggers activation of the caspase cascade, leading to cellular apoptosis.

In addition to apoptosis, the Fas/FasL pathway is also involved in the initiation of other cellular responses. These include maintenance of immune homeostasis, cell migration, and control of cancer cell invasiveness through regulation of mitogen-activated protein kinase and nuclear factor kappa B activation (10, 11). Apart from that, the Fas signaling pathway has been shown to drive cancer stemness through various mechanisms, such as activation of the ERK-JAG1 axis and the type I interferon/STAT1 axis (12, 13). While these studies have implicated *FAS* in cancer progression and response to therapy, studies exploring *FAS* gene expression and its potential role as a prognostic biomarker in lung cancer, particularly in NSCLC, are limited.

Given the important role of the Fas receptor in various aspects of oncogenesis, variations in the FAS gene have been shown to influence the risk and prognosis of many cancers (14–19). These effects are thought to be due to differential expression of *FAS* and its co-expressed genes in tumor cells compared with normal cells. However, the expression, prognostic significance and molecular interactions of *FAS* in lung cancer have not been systematically studied. We hypothesize that *FAS* gene expression is significantly altered in lung cancer compared to normal tissue and that genetic alterations, such as promoter methylation and mutations, could influence disease progression. However, the exact alterations are often not well-understood. For example, while FAS downregulation has been reported in lung cancer, there are also studies that show that can promote lung cancer growth *in vivo* (20, 21). The availability of genetic data in online tumor databases could provide useful information on the characteristics and potential role of *FAS* gene expression as a prognostic biomarker in cancers. Further, the potential modulation of *FAS* expression by patient characteristics such as age and smoking status in lung cancer context requires more detailed investigation. The clinical implications of understanding *FAS* expression in lung cancer are significant. If *FAS* expression and alterations are shown to have a prognostic impact, this could inform the development of new therapeutic approaches targeting the *FAS* pathway. Therefore, in this study, we performed data mining in online tumor databases to better understand the expression, prognostic significance and molecular interactions of *FAS* in lung cancer. This study aims to better understand the expression patterns, promoter methylation, genomic alterations, and potential protein-protein interactions of *FAS* in lung cancer, which could provide new insights into its role as a prognostic biomarker.

## 2 Materials and methods

### 2.1 Sample selection and preprocessing

All data used in this study were taken directly from publicly available databases and no additional pre-processing steps were performed. Sample selection criteria (i.e., inclusion of cancerous or normal tissue) were provided by the respective databases (e.g., TCGA, GEO, and Human Protein Atlas) and included data that passed the quality controls provided by the curators of the databases. We did not apply any specific inclusion or exclusion criteria beyond those specified in the databases. As all samples analyzed were from human subjects, biological replicates were not included in the analysis.

### 2.2 Gene expression analysis

The mRNA expression of FAS in human cancers was studied using GENT2 (http://gent2.appex.kr/gent2/), which extracts microarray data from the NCBI GEO database (22). GENT2 compiles gene expression profiles across a wide range of cancer types, allowing for the exploration of differential expression patterns in large datasets. Its strengths lie in its large sample size and robust statistical processing, but it is limited by the dependency on microarray data, which can be subject to batch effects and platform-specific biases. Subsequently, differential expression of FAS between lung cancer and normal lung tissue was examined using GEPIA2 (http://gepia2.cancer-pku.cn/), which extracts data from The Cancer Genome Atlas (TCGA) (23). GEPIA2 is a web-based tool specifically designed for cancer gene expression profiling and survival analysis based on RNA-Seq data from TCGA and GTEx, offering statistical significance testing through analysis of variance (ANOVA) and t-tests with FDR correction to control for multiple comparisons. Subgroup analysis of the TCGA expression data in different clinicopathological features was performed using UALCAN (http://ualcan.path.uab.edu) (24). UALCAN is a comprehensive, user-friendly platform for investigating cancer omics data with a focus on subgroup analysis based on clinicopathological parameters, using data from TCGA. The Human Protein Atlas database (https://www.proteinatlas.org/) was used to examine gene expression at the protein level through immunohistochemistry images (25).

### 2.3 Promoter methylation analysis

To determine whether the differential gene expression was driven by promoter methylation, the methylation level of FAS in lung cancers and paired normal tissues were compared using the UALCAN database. Methylation analysis in UALCAN used TCGA level-3 data processed through beta-values ranging from 0 to 1, where values closer to 0 indicate unmethylated CpG sites and values closer to 1 indicate fully methylated sites. Statistical comparisons between tumor and normal tissues were performed using a two-sample t-test, with correction for multiple comparisons using FDR.

### 2.4 Mutation and copy number alteration analysis

The presence and characteristics of FAS gene alterations, including mutations and copy number alterations, were analyzed using cBioPortal (https://www.cbioportal.org/), which contains information on various types of cancer genomics data (26). cBioPortal aggregates data from multiple sources, including TCGA and other cancer genomics projects. Mutational data are derived from whole-exome and whole-genome sequencing, and copy number alterations (CNAs) are identified using GISTIC 2.0 algorithms. Statistical analysis was performed using Fisher’s exact tests for comparing mutations.

### 2.5 Protein-protein interaction analysis

The protein-protein interaction networks of FAS were then reconstructed using the STRING (http://string.embl.de/) (27) and GeneMANIA (https://genemania.org/) (28) databases. STRING integrates known and predicted protein-protein interactions from multiple sources, including experimental data, computational prediction methods, and text mining, with interaction confidence scores based on the strength of evidence. GeneMANIA provides predictions using functional genomics data, including co-expression, colocalization, and physical interaction data. Both platforms employ machine-learning algorithms to predict novel interactions, but predictions can sometimes be prone to false positives or depend on incomplete datasets. STRING compiles data on protein-protein interactions from multiple sources and makes computational predictions to obtain a comprehensive global network of the interactions, whereas GeneMANIA uses extensive genomic and proteomic data to predict protein-protein interactions.

### 2.6 Co-expression analysis

Genes co-expressed with FAS are identified using GeneMANIA and UALCAN. GeneMANIA uses a combination of Pearson correlation coefficients and other statistical methods to identify genes that show similar expression patterns, which are then displayed in a network. UCSC Xena (https://xenabrowser.net/heatmap/) was then used to generate a correlation heat map with TCGA datasets to visualize the data (29). UCSC Xena applies Pearson correlation to measure the strength of co-expression between *FAS* and its associated genes, with statistical significance provided directly by the database.

### 2.7 Pathway analysis

Pathways involving FAS and the most frequently coexpressed genes were analyzed using Enrichr (https://maayanlab.cloud/Enrichr/), a gene set enrichment analysis tool, with default parameters (30). A Fisher’s exact test was used to evaluate the enrichment of gene sets within biological pathways, adjusting for multiple testing using the Benjamini-Hochberg method. GO terms are categorized into biological processes, molecular functions, and cellular components, and significance is determined through odds ratios and combined scores, which take into account both the magnitude of enrichment and significance. Potential limitations include reliance on existing annotations, which may not fully capture the complexity of gene interactions. Based on the GO terms, the input genes were categorized into biological processes, molecular functions, and cellular components.

### 2.8 Survival analysis

The prognostic significance of FAS in lung cancer was assessed using the OSluca web server (http://bioinfo.henu.edu.cn/LUCA/LUCAList.jsp), which performs hazard ratio (HR) analysis of data from various datasets, such as TCGA and GEO (31). Kaplan-Meier survival analysis was performed, along with log-rank tests, to determine the statistical significance of survival differences between groups based on *FAS* expression levels. The HR and 95% confidence intervals are provided for each dataset, and multiple comparisons are controlled using FDR correction. Results are pooled across datasets when appropriate to improve the statistical power of the analysis. HRs from the eligible datasets were then combined to estimate the impact of FAS gene expression on overall survival of lung cancer patients.

### 2.9 Protein expression

Data on FAS gene expression were retrieved from the Human Protein Atlas. This dataset comprised 578 samples from various tissues, with associated metadata including age, sex, and specific tissue type for each sample. The normalized transcripts per million (nTPM) values for FAS gene expression were extracted from the dataset. Immunohistochemistry images from the Human Protein Atlas were reviewed to confirm FAS protein expression across various tissues.

### 2.10 *In vitro* validation

Expression of *FAS* in lung cancer cell lines was examined using qRT-PCR. H1299, H1993, A549 and the normal bronchial epithelial cell line HBE were purchased from Shanghai Zhongqiao Xinzou Biotechnology Co., Ltd. and cultured in DMEM medium (Solarbio, Beijing, China) with 10% FBS and 1% penicillin-streptomycin. TRIzol reagent (from Invitrogen, Carlsbad, CA, USA) was used for total RNA extraction and RNA was transcribed into cDNA using ReverTra Ace qPCR RT Master Mix with gDNA Remover Kit. The qRT-PCR was performed using SYBR Premix Ex Taq II on the Mx3005P quantitative real-time fluorescence PCR system (from Stratagene, San Diego, CA, USA), and *GAPDH* was selected as the endogenous control for mRNA. The primer sequences are *FAS*, forward 5’-TCT GGT TCT TAC GTC TGT TGC-3’, reverse 5’-CTG TGC AGT CCC TAG CTT TCC-3’; *GAPDH*, forward 5’-GGA GCG AGA TCC CTC CAA AAT-3’, reverse 5’-GGC TGT TGT CAT ACT TCT CAT GG-3’. The reaction conditions were as follows: pre-denaturation at 95°C for 10 minutes, denaturation at 95°C for 5 seconds, annealing at 60°C for 30 seconds, for a total of 45 cycles. The target genes and the internal reference gene were amplified for each sample. Each sample group included three replicate wells. Data analysis was performed using the 2^(-ΔΔCt)^ method.

## 3 Results

### 3.1 Gene expression analysis

**Figure 1:**
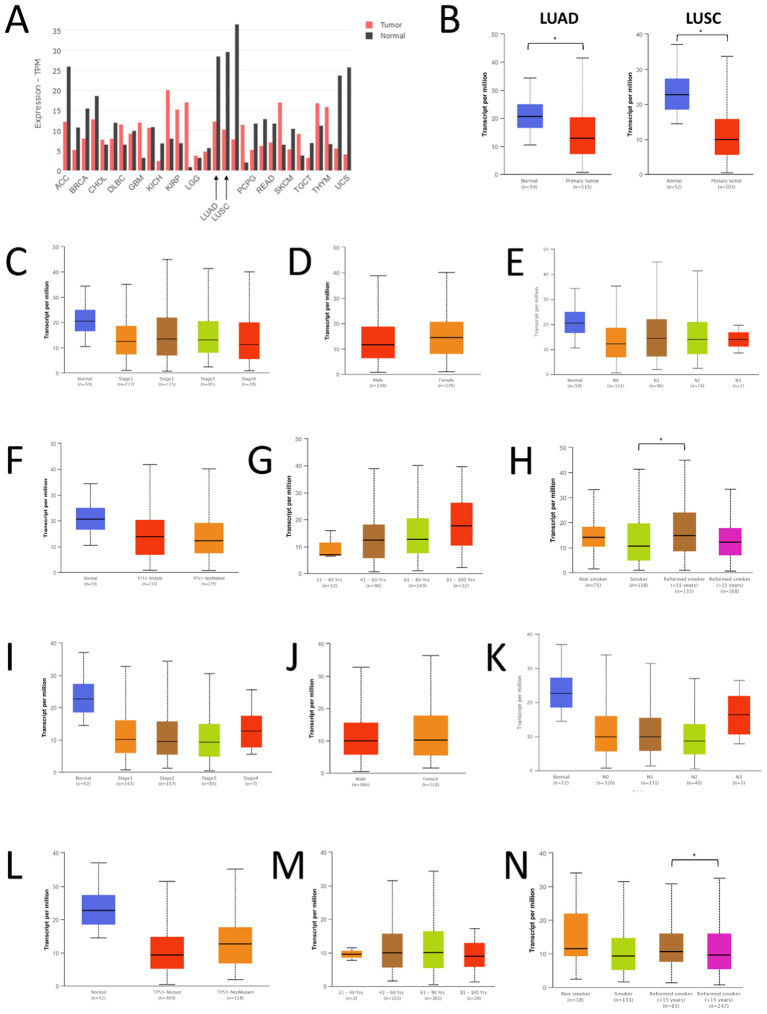
Expression of FAS in lung cancer and normal tissues. **(A)** Expression data from GEPIA. **(B)** Expression data from UALCAN. **(C)** Expression of FAS in different stages of LUAD. **(D)** Expression of FAS in men and women with LUAD. **(E)** Expression of FAS in LUAD patients with different nodal metastasis status. **(F)** Expression of FAS in LUAD patients with different TP53 mutations. **(G)** Expression of FAS in LUAD patients with different ages. **(H)** Expression of FAS in LUAD patients of different smoking status. **(I)** Expression of FAS in different stages of LUSC. **(J)** Expression of FAS in men and women with LUSC. **(K)** Expression of FAS in LUSC patients with different nodal metastasis status. **(L)** Expression of FAS in LUSC patients with different TP53 mutations. **(M)** Expression of FAS in LUSC patients with different ages. **(N)** Expression of FAS in LUSC patients of different smoking status. * Statistically significant (P<0.05).

Using GENT2, data on FAS gene expression were available for the GPL570 and GPL96 platforms. For both platforms, gene expression of FAS was found to be significantly altered in several cancer types (see **Supplementary Table 1**). In lung cancer, the expression of FAS was found to be significantly downregulated (P < 0.001 and log2FC = -0.569 for GPL570; P < 0.001 and -0.263 for GPL96). Expression data in GEPIA also showed that the expression of FAS was lower in tumor tissues compared with normal lung tissues in both LUAD (TPM =12.17 in tumor tissues and 28.41 in normal tissues) and LUSC (TPM =10.16 in tumor tissues and 29.52 in normal tissues) (**Figure 1A**). A similar observation was also found in the UALCAN database (P=5.61×10-8 for LUAD, P=5.75×10-12 for LUSC; **Figure 1B**). At the protein level, data from The Human Protein Atlas showed that in a sample of 578 lung cancer samples, the average nTPM of FAS was 21.7 (range: 5.2-67.2, median: 19.1).

We also performed a subgroup analysis of the expression of FAS in TCGA samples using UALCAN. In all stages of LUAD, the expression of FAS was lower than in normal tissues (**Figure 1C**). However, no significant difference was found between the different stages of cancer (P < 0.01). Similarly, no significant difference in FAS expression was observed between men and women (**Figure 1D**), different nodal metastasis status (**Figure 1E**), and different TP53 mutants (**Figure 1F**). Interestingly, when stratified by patient age, it was found that the older the patients, the higher the expression of FAS in general, although the difference between the different age groups was not statistically significant (**Figure 1G**). Similarly, there was no significant difference in FAS gene expression between nonsmokers and smokers, but former smokers (who had quit smoking for less than 15 years) had higher FAS gene expression than current smokers (**Figure 1H**, P = 0.019).

In LUSC, a similar observation was found for different cancer stages (**Figure 1I**), sex (**Figure 1J**), nodal metastasis status (**Figure 1K**), and TP53 mutation (**Figure 1L**). There was also no difference in the expression of FAS among different age groups, but the expression was more constant (i.e., showing no trend of increasing expression with increasing age) (**Figure 1M**). Regarding smoking status, there was also no significant difference in FAS gene expression between nonsmokers and smokers (**Figure 1N**). However, former smokers who had quit smoking for less than 15 years had significantly higher FAS expression than those who had quit smoking for more than 15 years (P < 0.01).

### 3.2 Promoter methylation analysis

**Figure 2:**
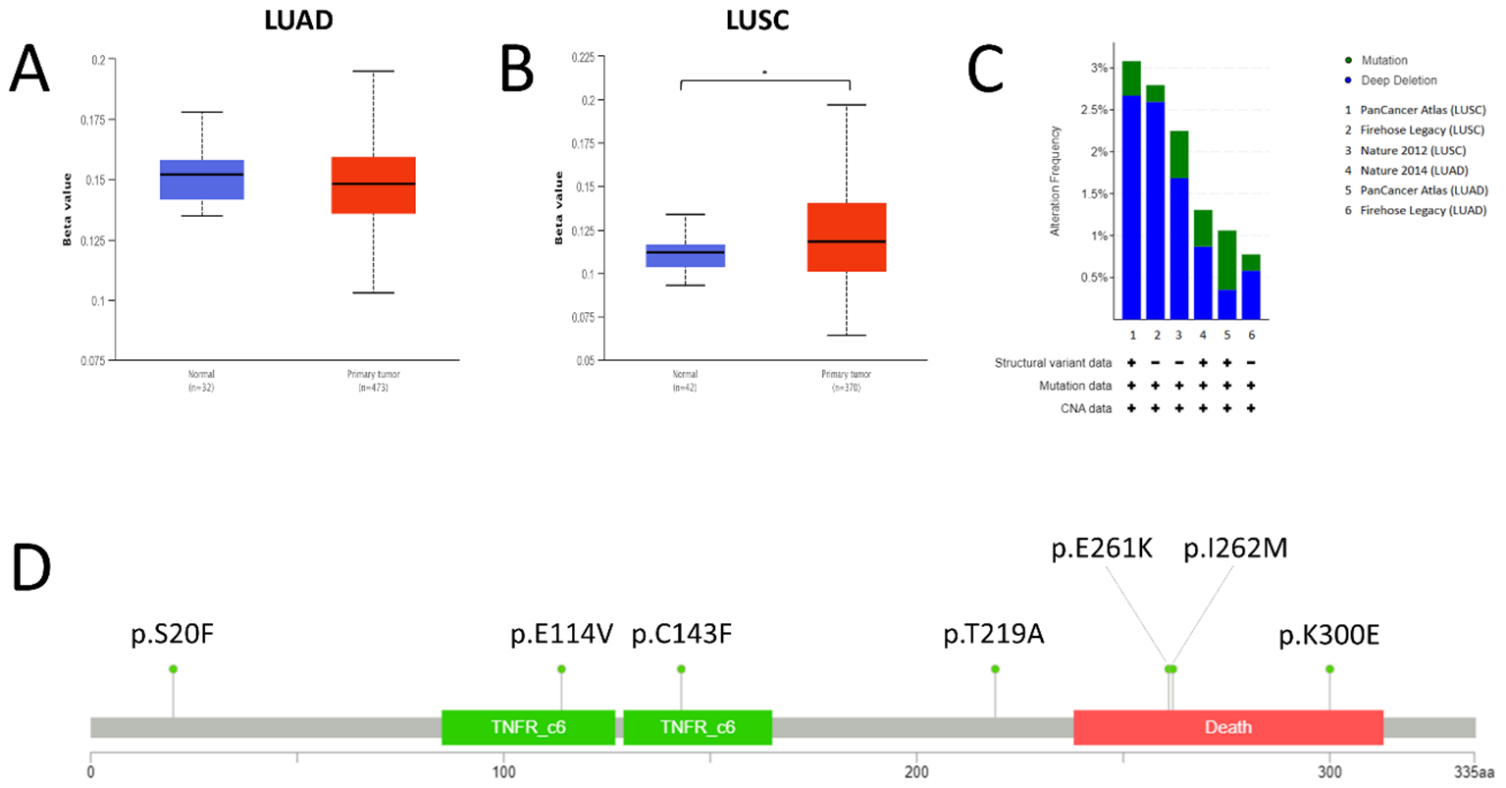
Promoter methylation, mutations and copy number status of FAS in lung cancer. **(A)** Promoter methylation level in LUAD. **(B)** Promoter methylation level in LUSC. **(C)** Prevalence of FAS alterations in different TCGA lung cancer datasets. **(D)** Lollipop diagram showing the location of FAS mutations in lung cancer. * Statistically significant (P<0.05).

The extent of FAS promoter methylation in TGCA samples was observed using UALCAN. In LUAD, the median beta-value of FAS promoter methylation in normal tissue is 0.152 (range: 0.135-0.178), whereas the value in tumor tissue is 0.148 (range: 0.103-0.195). There was no statistically significant difference between normal and LUAD tissue (P=0.149; **Figure 2A**). For LUSC, the median beta-value in normal and tumor tissues was 0.112 (range: 0.093-0.134) and 0.118 (range: 0.064-0.197), respectively. Beta-value was significantly higher in LUSC tissues than in normal tissues (P < 0.001; **Figure 2B**).

### 3.3 Mutation and copy number alteration analysis

We used cBioportal to investigate the prevalence and types of genomic alterations of the FAS gene in lung cancer patients. Information was available from a total of six TCGA datasets (Firehose Legacy, Nature 2014 and PanCancer Atlas for LUAD; Firehose Legacy, Nature 2012 and PanCancer Atlas for LUSC). Overall, FAS alterations were found in 46 (1.9%) of the 2478 samples (**Figure 2C**). Specifically, 36 (1.45%) of the samples had deep deletions and 10 (0.40%) had missense mutations (**Table 1**; **Figure 2D**). In LUAD, the missense mutations included p.E114V (N=3) and p.C143F (N=1) in the TNFR-like cysteine-rich domain, p.E261K (N=1) and p.K300E (N=1) in the death domain. In contrast, for LUSC, mutations included p.I262M (N=1) in the death domain and p.S20F (N=2) and p.T219A (N=1) in the non-domain region of the protein product.

**Table 1:** Prevalence of FAS alterations in lung cancer.

Dataset	Prevalence	
	Missense mutations	Deep deletion
LUSC PanCancer Atlas	2/487 (0.41%)	13/487 (2.67%)
LUSC Firehose Legacy	1/501 (0.20%)	13/501 (2.59%)
LUSC Nature 2012	1/178 (0.56%)	3/178 (1.69%)
LUAD Nature 2014	1/230 (0.43%)	2/230 (0.87%)
LUAD PanCancer Atlas	4/566 (0.71%)	2/566 (0.35%)
LUAD Firehose Legacy	1/516 (0.19%)	3/516 (0.58%)
Combined	10/2478 (0.40%)	36/2478 (1.45%)

### 3.4 Protein-protein interaction analysis

**Figure 3:**
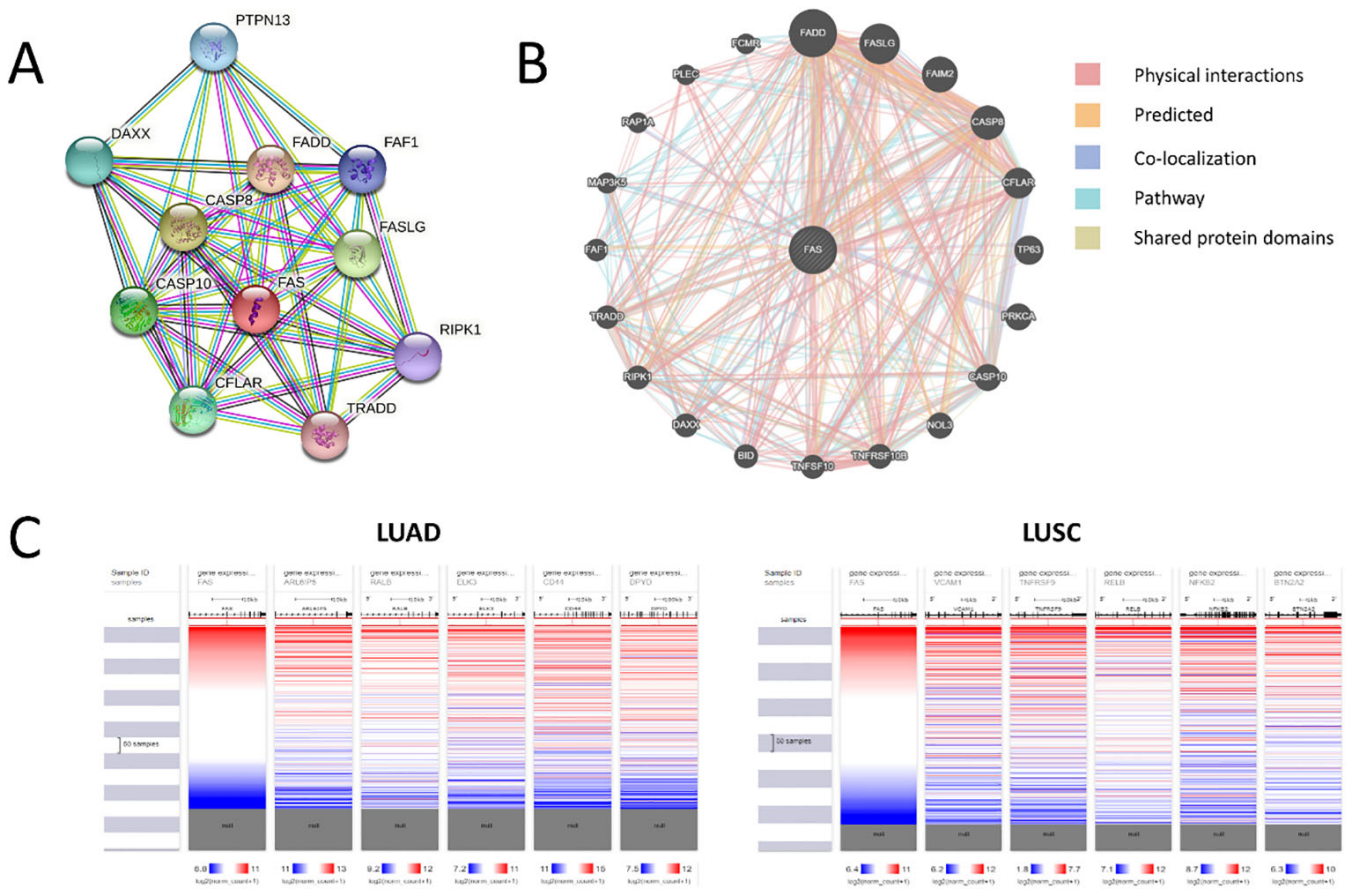
Protein-protein and genetic interactions of FAS. **(A)** Protein-protein interaction as predicted using STRING. **(B)** Overall protein-protein interaction network of FAS. **(C)** Correlation heat maps for top three co-expressed genes.

STRING analysis scored the protein-protein interaction using a score from 0 to 1, where 1 represents the highest probability that the interaction is true based on current evidence. Several proteins were shown to interact with FAS, namely FASLG (score = 0.999), CASP8 (score = 0.999), FADD (score = 0.999), CASP10 (score = 0.997), CFLAR (score = 0.996), DAXX (score = 0.995), PTPN13 (score = 0.995), FAF1 (score = 0.992), RIPK1 (score = 0.985), TRADD (score = 0.983) (**Figure 3A**). GeneMANIA, on the other hand, categorizes related genes into several categories, namely (1) physical interaction (protein-protein interaction), (2) shared protein domains, (3) colocalization (when genes are expressed in the same tissue or proteins are found in the same location), (4) pathway (two proteins are related when they are involved in the same signaling pathway), and (5) predicted protein interactions. For physical interaction, FAS has been shown to interact with BID, CASP10, CASP8, CFLAR, DAXX, FADD, FAF1, FAIM2, FASLG, MAP3K5, NOL3, PLEC, PRKCA, RAP1A, RIPK1, TNFRSF10B, TNFSF10, TP63, and TRADD. In addition, FADD, CASP8, CFLAR, CASP10, NOL3, TNFRSF10B, RIPK1, and TRADD shared protein domains with FAS. Proteins colocalizing with FAS include FASLG, CASP8, TP63, FADD, PRKCA, BID, DAXX, MAP3K5, and RAP1A. Besides, proteins that participate in the same signaling pathway as FAS include FADD, FASLG, CASP8, CFLAR, CASP10, BID, DAXX, RIPK1, TRADD, FAF1, MAP3K5, FCMR, and FAIM2. Finally, FAIM2, NOL3, TNFRSF10B, TNFSF10, FADD, CASP8, DAXX, and FAF1 are predicted to interact with FAS. The overall interaction network of FAS, as generated by GeneMANIA, is shown in **Figure 3B**.

### 3.5 Co-expression analysis

Analysis with GeneMANIA showed that CASP10, CFLAR, PRKCA, TNFRSF10B, TNFSF10, and TRADD are frequently co-expressed with FAS. Specifically for LUAD, UALCAN revealed 11 genes whose expression correlates with that of FAS, namely ARL6IP5 (Pearson’s coefficient, r = 0.61), RALB (r = 0.56), ELK3 (r = 0.53), CD44 (r = 0.52), DPYD (r = 0.52), GLIPR1 (r = 0.52), DAPP1 (r = 0.51), AIM1 (r = 0.51), LHFPL2 (r = 0.5), CFLAR (r = 0.5), and MDFIC (r = 0.5). On the other hand, in LUSC, 90 co-expressed genes were found, with the top 10 being VCAM1 (r = 0.72), TNFRSF9 (r = 0.69), RELB (r = 0.69), NFKB2 (r = 0.68), BTN2A2 (r = 0.67), BIRC3 (r = 0.67), SH2B3 (r = 0.67), PKDCC (r = 0.67), ZBTB46 (r = 0.66), and JAK2 (r = 0.66) (for the full list, please see **Supplementary Table 2**). Correlation heat maps generated by UCSC Xena showed for the top five co-expressed genes in LUAD and LUSC are shown in **Figure 3C**.

### 3.6 Pathway analysis

Gene set enrichment analysis confirmed that FAS and its most frequently co-expressed genes are involved in apoptosis-related pathways. Specifically, in the biological processes category, FAS and its co-expressed genes were found to be involved most predominantly in negative regulation of extrinsic apoptotic signaling pathway via death domain receptors (GO:1902042, P=2.53×10-18, adjusted P=7.15×10-16, OR=1725.2, combined score=69899.68). In terms of molecular functions, the genes were found to participate mainly in ubiquitin protein ligase binding (GO:0031625, P=5.937×10-6, adjusted P=1.049×10-4, odds ratio=50.39, combined score=606.4). The genes are also implicated in death-inducing signaling complex (GO:0031264, P=3.78×10-24, adjusted P=5.29×10-23, odds ratio=46641, combined score=2515479.09) in terms of cellular components.

### 3.7 Survival analysis

The OSluca web server contains survival data from 26 datasets. Eleven of the datasets had a hazard ratio (HR) value greater than 1.0 (indicating poor prognosis), although 10 of these were without statistical significance. Only the GSE68465 dataset showed borderline statistical significance at P=0.042 (HR =1.348, 95% CI=1.012-1.797). The remaining 15 datasets, which had an HR value of < 1.0, also did not reach statistical significance. Pooled results from all these datasets suggest that FAS expression has an HR of 1.302 (95% CI=0.935-1.139) (P=0.530), indicating a lack of prognostic significance.

### 3.8 Protein expression

The expression levels of the FAS protein across various lung cancer samples were investigated. From the dataset that comprised 578 samples, nTPM values of FAS protein in lung cancer samples ranged from 5.2 to 67.2. The average nTPM value across all samples was 21.7, with a median nTPM of 19.1. The standard deviation of the nTPM values was 9.5, indicating variability in FAS expression among the samples (**Supplementary Table 3**).

### 3.9 *In vitro* validation

**Figure 4:**
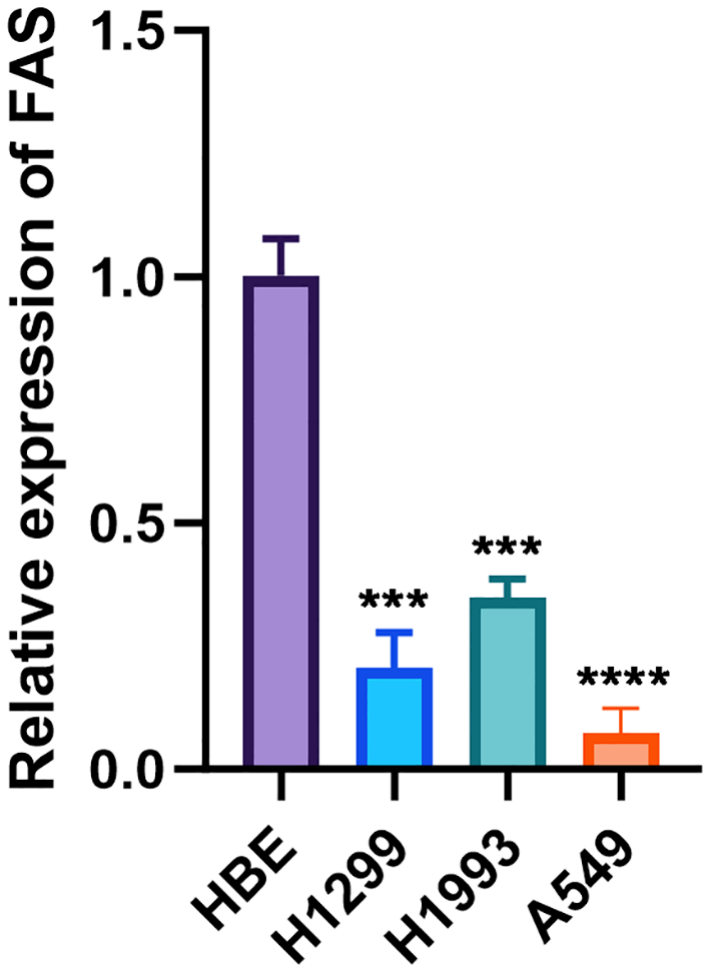
Relative expression of FAS in lung cancer cell lines and normal bronchial epithelial cells. The bar graph shows the relative expression levels of FAS in normal bronchial epithelial cell line (HBE) and lung cancer cell lines (H1299, H1993, A549). Data are presented as mean ± SD. Statistical significance is indicated by asterisks: ***p < 0.001, ****p < 0.0001 compared to HBE.

The expression levels of the *FAS* were investigated in three lung cancer cell lines and a normal cell line. The relative expression of *FAS* in lung cancer cell lines (H1299, H1993, A549) was significantly lower than the normal bronchial epithelial cell line (HBE), with *GAPDH* serving as the internal control. The difference was statistically significant (p < 0.001 for H1299 and H1993, p < 0.0001 for A549; **Figure 4**; **Supplementary Table 4**).

## 4 Discussion

Apoptosis, a tightly regulated process of programmed cell death, plays a crucial role in the pathogenesis of lung cancer. However, the specific characteristics (in terms of expression, mutational and epigenetic profiles, and protein and gene interactions) of apoptosis-related genes such as FAS and their prognostic significance are not well understood. In this study, we sought to clarify these unknowns through an extensive search of online databases. As interpretation of the impact of a genetic variant may vary depending on the specific databases, it is important to consider multiple sources of information when investigating the clinical and biological characteristics of a gene. We therefore searched multiple databases (e.g., GENT2, GEPIA2, and UALCAN for gene expression; STRING and GeneMANIA for protein-protein interaction; etc.) to obtain a conclusive result about the role of FAS in lung cancer. These databases were chosen due to their extensive and curated datasets, wide adoption in cancer research, and their ability to provide different layers of information. For instance, GENT2 and GEPIA2 integrate data from large consortia like TCGA and provide a detailed breakdown of gene expression across cancers, whereas STRING and GeneMANIA focus on elucidating protein and gene interaction networks, helping to contextualize gene function in a broader biological network. However, GENT2 and GEPIA2 are limited by their reliance on bulk RNA sequencing and microarray data, which may obscure cell-type-specific expression patterns, and they may not capture transcript variants or post-transcriptional modifications. UALCAN was selected because of its user-friendly interface and comprehensive analysis of clinicopathological features, which allows for meaningful subgroup analysis based on patient characteristics such as smoking status, age, and tumor stage. A limitation of UALCAN is its dependence on TCGA data, which, although extensive, may not be representative of all population demographics, and batch effects or data inconsistencies across studies can influence the outcomes. STRING and GeneMANIA focus on interaction networks, but STRING relies heavily on computational predictions and text mining, which can introduce false positives, while GeneMANIA’s predictions are not always experimentally validated, and both tools may omit less well-characterized interactions. Nevertheless, the use of multiple databases allowed us to ensure robustness of our findings and reduce potential biases inherent in any single database (32). Nonetheless, it is important to note that the use of in silico data mining limits direct biological validation of our findings, and experimental confirmation is needed in future studies. Nevertheless, the findings of this study may provide valuable insight into the role of FAS in the pathogenesis of lung cancer.

One of the most important findings of this work is that FAS was significantly downregulated in lung cancer, both in LUAD and LUSC. The downregulation of FAS was also validated in our *in vitro* analysis in three lung cancer cell lines, when compared against the normal bronchial epithelial cell line HBE. However, it is important to clarify that while this downregulation was statistically significant, its clinical implications remain unclear, as no strong association with patient prognosis was observed. In LUAD, we also observed that former smokers (who had quit smoking for less than 15 years) had higher FAS gene expression than current smokers. This observation is not surprising, because smoking cessation is known to reverse smoking-induced DNA methylation changes (33). Thus, smoking cessation can restore the expression of FAS to levels that are close to those of nonsmokers. However, this finding should be interpreted with caution as it does not account for confounding variables such as the extent and duration of smoking exposure before quitting, which can significantly influence gene expression patterns. Future studies should aim to collect more detailed smoking history data to better understand these interactions. An interesting finding was noted in LUSC, where former smokers who had quit smoking for less than 15 years had significantly higher FAS expression than those who had quit smoking for more than 15 years. This observation is counterintuitive because, according to the logic above, the longer a person has quit smoking, the higher the FAS gene expression should be. However, this observation did not take into account the intensity and duration of smoking before quitting, which may also affect gene expression (34). In addition, gene expression may also be influenced by other factors such as age, sex, and other genetic and nongenetic factors that may interact with smoking status and affect the expression of FAS. Future studies are needed to clarify the underlying mechanisms and potential clinical implications of these findings.

At the protein level, we observed variability in the nTPM levels of FAS protein in the lung cancer samples, ranging from 5.2 to 67.2, suggesting considerable heterogeneity in FAS protein expression across tumor samples. While many samples have moderate FAS expression, some tumors have either significantly elevated or reduced levels of the protein. High FAS expression could indicate an increased capacity for apoptosis in some tumors, possibly serving as a mechanism for tumor suppression, while lower levels of FAS contribute to apoptosis evasion, facilitating tumor progression and resistance to cell death. This differential expression could also reflect differences in the molecular subtype of lung cancer, the tumor microenvironment or the influence of external factors such as smoking or previous treatments. Understanding the causes and consequences of this variability is critical as it may provide insight into tumor behavior, prognosis and therapeutic response, particularly in relation to therapies targeting apoptotic pathways. Further studies are needed to determine how FAS protein levels correlate with clinical outcomes and treatment efficacy in lung cancer.

Given the significantly different expression levels between lung tumor tissues and their normal counterparts, we next sought to determine whether there was a significant difference in the methylation levels of cancerous and noncancerous tissues of the lung. We did not find a statistically significant difference between normal and LUAD tissues. However, beta-value was significantly higher in LUSC than in normal lung tissues, indicating greater methylation in cancerous tissues. Nevertheless, both normal and LUSC tissues have low levels of DNA methylation (beta-value < 0.3), suggesting that methylation is unlikely to play a dominant role in affecting the expression of FAS. Other factors, such as noncoding RNAs, silencers, enhancers, and transcription factors, might also affect FAS expression (35–37). Indeed, FAS is known to be transcriptionally regulated by members of the p53 family (38), and several silencer and enhancer sequences in the FAS gene have also been identified since the 1990s (39). More recently, antisense RNA of FAS, FAS-AS1 or Saf, has been identified, and is thought to affect the expression of FAS and shown to have functional effects (40, 41). Thus, further studies are needed to investigate the role of these factors in affecting FAS expression. It will also be important to examine whether the methylation status of FAS is tissue-specific and whether certain lung cancer subtypes exhibit unique epigenetic signatures that could provide therapeutic targets.

We have also shown that alterations in FAS are a rare event in lung cancer, occurring in 1.9% of all samples. Deep deletions represent the predominant form of FAS alterations. FAS deletions have been observed in many cancers, including prostate, colorectal, and gastric cancers, but the exact prevalence is not well known because previous studies have typically used small sample sizes (42–44). FAS deletions have been associated with impaired apoptosis, which may serve as an important mechanism of carcinogenesis (44). In addition to small sample sizes, discrepancies in the reported frequency of FAS deletions may also arise from differences in methodologies, such as the use of different sequencing platforms or variant calling algorithms, which can affect the detection of deletions. Furthermore, variations in patient populations, including differences in tumor stage, histological subtype, or demographic factors like age and smoking status, could contribute to variability in FAS deletion prevalence across studies. *In vivo* studies found that deletion of FAS can increase the size and number of intestinal adenomas in mice (45). Another study showed that deletion of FAS, when accompanied by deletion of PTEN, is associated with poor prognosis in hormone-refractory prostate cancer (43). However, deletion of FAS has been shown not to affect its expression (42). It is also difficult to determine whether these genomic changes have a significant clinical impact in lung cancer, as the frequency of these FAS alterations was low. Therefore, the significance of FAS deletion in carcinogenesis requires further research.

We also identified missense mutations in 0.40% of lung cancer samples. The mutations present in LUAD are different from those in LUSC. However, the small number of affected samples does not allow us to reliably determine whether the findings can be interpreted as different mechanisms of carcinogenesis in the two lung cancer subtypes. The functional consequences of these variants have not been thoroughly studied, so it is not known whether they play a driving role in lung cancer. Nevertheless, the p.E261K mutation found in LUAD and the p.I262M mutation found in LUSC, both of which result in amino acid changes in the death domain of the Fas protein, have been linked to autoimmune lymphoproliferative syndrome (46–48). *In vitro* studies revealed that the p.E261K mutation can impair the process of reorganization of Fas into large protein islands and also has a dominant-negative property that adversely affects the normal wild-type Fas during the formation of the Fas-FADD signaling complex (47, 48). While these findings suggest potential functional effects of these mutations, the lack of statistical significance and the small number of cases prevent us from making broad generalizations. Additional functional studies are needed to determine whether these mutations have oncogenic or tumor-suppressive roles in lung cancer.

Genes and proteins often engage in various forms of molecular interactions, such as gene-gene and protein-protein interactions, to perform their biological functions (49, 50). Therefore, it is important to understand these interactions to decipher the complexity of biological systems. We have performed protein-protein interactions and genetic co-expression analyses to identify proteins and genes that may interact with FAS. Perhaps not surprisingly, many of the identified genes/proteins, such as FASLG, CASP8, FADD, CASP10, BID, TRADD, and CFLAR, are involved in the apoptotic process. This finding supports the hypothesis that FAS and its associated genes and proteins play a key role in regulating apoptosis in lung cancer. However, the lack of mechanistic studies in lung cancer cells limits our ability to determine the functional importance of these interactions. Investigating these interactions *in vitro* or *in vivo* could provide deeper insights into their relevance in lung cancer progression (51). It is interesting to note that 90 genes were significantly co-expressed in LUSC, whereas only 11 genes were significantly co-expressed in LUAD, which may reflect the differences in molecular and cellular processes involved in the two lung cancer subtypes. The higher number of co-expressed genes in LUSC suggests that the molecular networks and signaling pathways in LUSC are more complex and interconnected than those in LUAD. Indeed, a recent study also demonstrated that many cancer-related signaling pathways, including Notch, Hedgehog, Wnt, and ErbB pathways, were significantly overrepresented in LUSC compared to LUAD (52). Another possible explanation for this observation is that compared to LUAD, LUSC is more frequently associated with tobacco smoking, which can cause extensive genomic damage and activate many cellular signaling pathways, including inflammation and oxidative stress, that can further drive cancer development (52, 53). The involvement (or lack thereof) of these signaling pathways in oncogenesis may also contribute to the observed differences in co-expression between the two types of lung cancer. The clinical significance of these differences in co-expressed genes between LUAD and LUSC is not well understood and represents a future research direction. Further exploration into the functional consequences of these differences could reveal important subtype-specific therapeutic targets and guide personalized treatment strategies (54).

The lack of significant prognostic value for FAS in this study, despite it being downregulated in lung cancer, highlights an important aspect of cancer research — the importance of negative findings. While FAS plays a role in several types of cancer, its role in lung cancer may be more context-dependent or its prognostic significance may be overshadowed by other factors. This negative result is valuable because it prompts future research to consider more complex, multifactorial prognostic models that include additional markers or signaling pathways. It also suggests that the role of FAS in lung cancer may not be clear, so its interactions with other apoptotic or non-apoptotic metabolic pathways and its behavior under different conditions of the tumor microenvironment need to be further investigated (55). Negative results such as these help to refine research questions and focus on promising targets or combinations of biomarkers that could provide clinically meaningful prognostic information.

Despite successfully demonstrating the reduced expression of the FAS gene in lung cancer, survival analysis revealed no prognostic significance of the gene in lung cancer. This suggests that although downregulation of FAS is a common feature of lung cancer, it may not be a reliable predictor of disease outcome. This result was not consistent with previous findings in lung and other cancers, which showed a significant association between expression of FAS and cancer prognosis (56–60). However, it should be noted that previous studies on the prognostic significance of the FAS gene used small sample sizes, which may lead to inaccurate conclusions (61). Our in silico data mining combined survival data from 26 datasets, which greatly improved the statistical power required for accurate analysis. The lack of prognostic significance in our study may indicate that while FAS plays a role in tumor initiation, its downregulation may not be critical for disease progression or metastasis in lung cancer. One possible explanation for the limited prognostic significance of FAS is that although the gene plays a role in the early stages of lung cancer development, its expression may not be critical for tumor progression or metastasis. However, this postulation is not supported by several studies that showed that FAS can promote progression and metastasis in various cancers (10, 13, 62, 63). Nevertheless, none of these studies were conducted in lung cancer cells, and it remains unclear whether FAS plays a role in lung cancer cell progression and metastasis. Further *in vitro* and *in vivo* studies are needed to explore the role of FAS in lung cancer metastasis, particularly to assess whether its downregulation affects the invasive potential of lung cancer cells. It is also possible that there are other confounding factors or co-occurring genes that affect lung cancer progression and patient survival, which could limit the prognostic significance of FAS expression in the cancer. The role of FAS and other factors in influencing lung cancer progression and metastasis deserves further investigation. Future studies should aim to investigate whether FAS expression in combination with other apoptotic markers could provide a more accurate prognostic model for lung cancer.

## 5 Conclusions

In conclusion, we have successfully characterized the role of FAS in lung cancer. Specifically, we have shown that FAS is significantly downregulated in lung cancer and characterized its mutational and methylation profiles. We also identified its protein-protein interactions and co-expressed genes and reconfirmed the important role of FAS and its co-expressed genes in apoptosis-related pathways. Finally, we have shown that despite the above observations, the prognostic significance of FAS in lung cancer is limited. The clinical implications of FAS downregulation, alterations, and molecular interactions, as well as the differences between LUAD and LUSC in these features, remain to be investigated. Thus, there is a need for more comprehensive and integrative approaches to understand the molecular and cellular mechanisms of FAS that drive lung cancer progression. Future studies should focus on functional analyses of FAS and its mutations in lung cancer cells to better understand how downregulation of FAS contributes to apoptosis evasion. In addition, it will be important to investigate the role of non-coding RNAs, transcription factors and other regulatory elements that may influence FAS expression. Further research should also investigate the potential of FAS as part of a biomarker panel in combination with other apoptotic genes for a more accurate prognosis. Finally, *in vivo* studies are needed to assess whether modulation of FAS expression could have therapeutic potential in lung cancer, either as a direct target or in combination with existing treatments.

## Data Availability

The original contributions presented in the study are included in the article/**Supplementary Material**. Further inquiries can be directed to the corresponding author.
