# Supplementary material for: FAS gene expression, prognostic significance and molecular interactions in lung cancer
Source: Front Oncol. 2024 Oct 2;14:1473515. doi: 10.3389/fonc.2024.1473515 (PMC11479862; doi:10.3389/fonc.2024.1473515)
Supplement: Supplementary file 1 [file Table1.docx]

Supplementary Table 1: *FAS* gene expression in normal and tumor tissues of the lung – GENT2 database

| **Tissue** | **GPL570 platform** | | **GPL96 platform** | |
| --- | --- | --- | --- | --- |
|  | **P value** | **Log2FC** | **P value** | **Log2FC** |
| All | <0.001 | -0.266 | <0.001 | -0.149 |
| Adipose | 0.023 | -0.439 | NA | NA |
| Adrenal gland | 0.207 | -0.213 | 0.002 | -0.632 |
| Bladder | 0.820 | 0.027 | 0.711 | -0.087 |
| Blood | <0.001 | -1.244 | <0.001 | -1.722 |
| Bone marrow | NA | NA | <0.001 | 0.608 |
| Bone | 0.339 | -0.235 | NA | NA |
| Brain | <0.001 | 0.845 | <0.001 | 0.413 |
| Breast | <0.001 | -0.376 | <0.001 | -0.520 |
| Cartilage | NA | NA | 0.729 | -0.198 |
| Cervix | 0.798 | -0.043 | 0.003 | -0.430 |
| Colon | <0.001 | -1.127 | <0.001 | -0.708 |
| Endometrium | 0.432 | -0.073 | NA | NA |
| Esophagus | 0.003 | -0.542 | 0.474 | -0.065 |
| Eye | NA | -1.221 | NA | NA |
| Gallbladder | 0.505 | -0.296 | NA | NA |
| Head and neck | <0.001 | -0.598 | NA | NA |
| Heart | NA | NA | <0.001 | 1.461 |
| Immune system | NA | NA | <0.001 | 0.708 |
| Joint | NA | NA | 0.013 | 0.256 |
| Kidney | <0.001 | 0.687 | 0.013 | -0.191 |
| Larynx | NA | NA | 0.919 | 0.085 |
| Liver | <0.001 | -0.715 | 0.412 | 0.085 |
| **Lung** | **<0.001** | **-0.569** | **<0.001** | **-0.263** |
| Lymph node | NA | -0.538 | NA | NA |
| Muscle | 0.373 | 0.900 | 0.670 | 0.246 |
| Oral | <0.001 | -0.837 | NA | NA |
| Ovary | <0.001 | -0.489 | <0.001 | -1.044 |
| Pancreas | 0.122 | 0.179 | <0.001 | 0.928 |
| Pharynx | 0.123 | 0.894 | 0.436 | 0.574 |
| Placenta | NA | NA | NA | NA |
| Prostate | 0.261 | -0.121 | 0.119 | 0.123 |
| Skin | <0.001 | -0.771 | <0.001 | -1.199 |
| Small intestine | 0.196 | 0.509 | NA | NA |
| Soft tissue | NA | NA | 0.005 | 0.665 |
| Spleen | 0.092 | 0.417 | NA | NA |
| Stomach | 0.238 | -0.103 | <0.001 | 0.225 |
| Teeth | 0.954 | -0.019 | NA | NA |
| Testis | 0.634 | 0.356 | <0.001 | -0.784 |
| Thyroid | <0.001 | 1.196 | 0.167 | 0.231 |
| Tongue | 0.017 | 0.561 | 0.559 | 0.118 |
| Urothelium | NA | NA | <0.001 | -0.770 |
| Uterus | 0.235 | 0.198 | 0.042 | -0.327 |
| Vagina | 0.818 | 0.080 | NA | NA |
| Vulva | 0.239 | 0.207 | NA | NA |
