# Supplementary material for: FAS gene expression, prognostic significance and molecular interactions in lung cancer
Source: Front Oncol. 2024 Oct 2;14:1473515. doi: 10.3389/fonc.2024.1473515 (PMC11479862; doi:10.3389/fonc.2024.1473515)
Supplement: Supplementary file 2 [file Table2.docx]

Supplementary Table 2: List of genes co-expressed with *FAS* in LUSC

| *VCAM1* (r = 0.72) | *ACHE* (r = 0.53) | *LCP1* (r = 0.51) |
| --- | --- | --- |
| *TNFRSF9* (r = 0.69) | *PARM1* (r = 0.53) | *FAM65C* (r = 0.51) |
| *RELB* (r = 0.69) | *ALDH2* (r = 0.53) | *FMNL3* (r = 0.51) |
| *NFKB2* (r = 0.68) | *CIITA* (r = 0.53) | *LGI2* (r = 0.51) |
| *BTN2A2* (r = 0.67) | *ETS1* (r = 0.53) | *NEURL3* (r = 0.5) |
| *BIRC3* (r = 0.67) | *RNF19A* (r = 0.53) | *ADAM8* (r = 0.5) |
| *SH2B3* (r = 0.67) | *NEDD1* (r = 0.53) | *RNF19B* (r = 0.5) |
| *PKDCC* (r = 0.67) | *TMEM150C* (r = 0.52) | *ANKRD29* (r = 0.5) |
| *ZBTB46* (r = 0.66) | *ERO1LB* (r = 0.52) | *TMEM200A* (r = 0.5) |
| *JAK2* (r = 0.66) | *LOC84856* (r = 0.52) | *CD86* (r = 0.5) |
| *UBD* (r = 0.66) | *UBA7* (r = 0.52) | *TIFA* (r = 0.5) |
| *TLR1* (r = 0.65) | *ERAP1* (r = 0.52) | *IRF1* (r = 0.5) |
| *IL2RG* (r = 0.63) | *C5orf56* (r = 0.52) | *LSAMP* (r = 0.5) |
| *IL15* (r = 0.63) | *BTN3A3* (r = 0.52) | *LITAF* (r = 0.5) |
| *INPP1* (r = 0.62) | *TRAF1* (r = 0.52) | *CSF2RB* (r = 0.5) |
| *IL21R* (r = 0.62) | *PRRX1* (r = 0.52) | *SLC12A7* (r = 0.5) |
| *SYNPO2* (r = 0.62) | *APOL3* (r = 0.52) | *PLEK* (r = 0.5) |
| *NFKBIE* (r = 0.62) | *NINJ1* (r = 0.51) | *SYTL3* (r = 0.5) |
| *ICAM1* (r = 0.61) | *CD40* (r = 0.51) | *EPSTI1* (r = 0.5) |
| *PNRC1* (r = 0.6) | *GCET2* (r = 0.51) | *INPP4A* (r = 0.5) |
| *BMP2K* (r = 0.59) | *ACHE* (r = 0.53) | *LCP1* (r = 0.51) |
| *SPIB* (r = 0.59) | *PARM1* (r = 0.53) | *FAM65C* (r = 0.51) |
| *JAK3* (r = 0.59) | *ALDH2* (r = 0.53) | *FMNL3* (r = 0.51) |
| *CLEC7A* (r = 0.59) | *CIITA* (r = 0.53) | *LGI2* (r = 0.51) |
| *NECAP2* (r = 0.59) | *ETS1* (r = 0.53) | *NEURL3* (r = 0.5) |
| *CDH23* (r = 0.59) | *RNF19A* (r = 0.53) | *ADAM8* (r = 0.5) |
| *SLC2A6* (r = 0.59) | *NEDD1* (r = 0.53) | *RNF19B* (r = 0.5) |
| *RFTN1* (r = 0.59) | *TMEM150C* (r = 0.52) | *ANKRD29* (r = 0.5) |
| *TNFRSF1B* (r = 0.58) | *ERO1LB* (r = 0.52) | *TMEM200A* (r = 0.5) |
| *SRGN* (r = 0.58) | *LOC84856* (r = 0.52) | *CD86* (r = 0.5) |
