# Supplementary material for: FAS gene expression, prognostic significance and molecular interactions in lung cancer
Source: Front Oncol. 2024 Oct 2;14:1473515. doi: 10.3389/fonc.2024.1473515 (PMC11479862; doi:10.3389/fonc.2024.1473515)
Supplement: Supplementary file 3 [file Table3.docx]

Supplementary Table 3: Normalized expression data of *FAS* gene from the Human Protein Atlas database

| **GTEx sample ID** | **Sample description** | **Normalized expression (nTPM)** |
| --- | --- | --- |
| GTEX-YFC4-1126-SM-5RQJN | 40-49 years, female | 67.2 |
| GTEX-1J8JJ-1126-SM-CL55P | 60-69 years, female | 62.9 |
| GTEX-147F4-0926-SM-5Q5EO | 50-59 years, male | 61.9 |
| GTEX-1QEPI-0826-SM-DTX9B | 60-69 years, female | 59.1 |
| GTEX-1N2DW-1126-SM-EVR59 | 50-59 years, male | 58.9 |
| GTEX-14BMV-0826-SM-73KXU | 50-59 years, male | 55.4 |
| GTEX-111VG-0726-SM-5GIDC | 60-69 years, male | 54.7 |
| GTEX-13OW7-0926-SM-5L3EX | 50-59 years, male | 54 |
| GTEX-RNOR-0726-SM-2TF5I | 50-59 years, female | 53.3 |
| GTEX-RVPV-1726-SM-2NKAQ | 60-69 years, male | 52.5 |
| GTEX-1LB8K-0826-SM-D4P41 | 50-59 years, male | 52.2 |
| GTEX-11ZUS-0126-SM-5EQM5 | 30-39 years, male | 51.6 |
| GTEX-1A8FM-0826-SM-793D5 | 50-59 years, female | 51 |
| GTEX-1RQED-1026-SM-EVYCT | 60-69 years, male | 50.8 |
| GTEX-1EKGG-1326-SM-7MXUM | 60-69 years, male | 50.2 |
| GTEX-17JCI-1526-SM-7IGOQ | 60-69 years, female | 49 |
| GTEX-X4EO-0926-SM-3P5Z2 | 60-69 years, female | 48.9 |
| GTEX-1E2YA-0926-SM-7MXUK | 50-59 years, male | 47.6 |
| GTEX-13OVL-0626-SM-5KM13 | 50-59 years, male | 47.2 |
| GTEX-1JJEA-1126-SM-CKZOC | 60-69 years, male | 46.1 |
| GTEX-11O72-1326-SM-5BC5A | 40-49 years, male | 46 |
| GTEX-11WQK-1226-SM-5GU5Z | 50-59 years, male | 45.7 |
| GTEX-117YW-0526-SM-5H11C | 50-59 years, male | 45.5 |
| GTEX-1MA7X-0626-SM-E9TJI | 60-69 years, male | 45 |
| GTEX-1KD4Q-0926-SM-CXZKI | 40-49 years, male | 44.5 |
| GTEX-16Z82-1026-SM-6M48A | 60-69 years, male | 43.3 |
| GTEX-12584-1426-SM-5EGJ9 | 40-49 years, male | 43.1 |
| GTEX-13O3O-0726-SM-5J1N7 | 60-69 years, female | 42.6 |
| GTEX-1LVAO-1226-SM-DIPGB | 60-69 years, male | 41.4 |
| GTEX-1F75W-0626-SM-7PC13 | 60-69 years, male | 40.6 |
| GTEX-ZDXO-1326-SM-57WBS | 60-69 years, male | 40 |
| GTEX-1H1CY-0626-SM-9KNVE | 60-69 years, female | 39.9 |
| GTEX-1B8L1-0626-SM-7EPHL | 60-69 years, male | 39.7 |
| GTEX-1C6VR-0626-SM-79OOY | 60-69 years, male | 39.7 |
| GTEX-1IOXB-1126-SM-CNNQ5 | 50-59 years, male | 39.2 |
| GTEX-1GZ2Q-0726-SM-9KNVV | 40-49 years, male | 38.5 |
| GTEX-1F6I4-1026-SM-7P8PS | 60-69 years, male | 38.2 |
| GTEX-1I1GP-0526-SM-ARL7G | 50-59 years, male | 37.7 |
| GTEX-1A8G6-0726-SM-73KV5 | 60-69 years, male | 37.6 |
| GTEX-13G51-0426-SM-5K7Z5 | 60-69 years, male | 36.6 |
| GTEX-1H1DG-1026-SM-9MQJS | 70-79 years, male | 36.6 |
| GTEX-ZDTS-1026-SM-4WAXS | 60-69 years, male | 36.5 |
| GTEX-1PWST-0826-SM-DTXF1 | 70-79 years, male | 36.5 |
| GTEX-1EMGI-1426-SM-7IGNQ | 60-69 years, male | 36.3 |
| GTEX-18A6Q-0826-SM-7KFRD | 60-69 years, male | 36.2 |
| GTEX-OXRO-0326-SM-33HBM | 60-69 years, female | 35.8 |
| GTEX-ZVT3-0926-SM-5GICK | 60-69 years, female | 35.8 |
| GTEX-131XH-0426-SM-5DUWU | 50-59 years, male | 35.4 |
| GTEX-SIU8-0926-SM-COH1X | 50-59 years, male | 35.4 |
| GTEX-1CB4J-1726-SM-79OOL | 60-69 years, male | 34.2 |
| GTEX-132NY-1226-SM-5PNVF | 60-69 years, male | 34.1 |
| GTEX-1EWIQ-0926-SM-7RHG7 | 70-79 years, female | 34.1 |
| GTEX-PVOW-1026-SM-2XCF9 | 40-49 years, male | 33.9 |
| GTEX-WYBS-1126-SM-3NMAM | 60-69 years, female | 33.9 |
| GTEX-1F88F-0926-SM-7RHH8 | 60-69 years, female | 33.9 |
| GTEX-145LU-0526-SM-5O9AT | 50-59 years, male | 33.7 |
| GTEX-N7MS-0926-SM-2HMIZ | 60-69 years, male | 33.3 |
| GTEX-SN8G-0926-SM-4DM5I | 50-59 years, female | 33.3 |
| GTEX-148VJ-0826-SM-5LU8V | 70-79 years, male | 33 |
| GTEX-1RNSC-1226-SM-E9U52 | 60-69 years, male | 33 |
| GTEX-17MF6-0826-SM-7LT8F | 40-49 years, male | 32.9 |
| GTEX-139UW-0226-SM-5K7WU | 60-69 years, male | 32.8 |
| GTEX-1PIIG-1826-SM-DTX7N | 30-39 years, male | 32.7 |
| GTEX-13OW6-0826-SM-5L3GA | 50-59 years, male | 32.6 |
| GTEX-15ER7-0926-SM-7KUMG | 20-29 years, female | 32.4 |
| GTEX-17F96-0626-SM-793CC | 70-79 years, male | 32.4 |
| GTEX-13JVG-1426-SM-5MR4W | 60-69 years, male | 32.3 |
| GTEX-13OVH-1026-SM-5J2NP | 70-79 years, male | 31.9 |
| GTEX-13OVJ-0726-SM-5KM1W | 50-59 years, female | 31.8 |
| GTEX-1IDJH-0726-SM-ARU7U | 70-79 years, male | 31.6 |
| GTEX-16GPK-1826-SM-7MGW5 | 60-69 years, male | 31.4 |
| GTEX-1C6WA-0726-SM-7IGQ5 | 60-69 years, male | 31.1 |
| GTEX-139TT-0726-SM-5K7XW | 60-69 years, male | 30.8 |
| GTEX-1HSKV-1126-SM-ADEIA | 60-69 years, male | 30.8 |
| GTEX-1KWVE-0426-SM-DHXJX | 30-39 years, male | 30.8 |
| GTEX-139YR-0926-SM-5LZYB | 50-59 years, male | 30.7 |
| GTEX-UPJH-0826-SM-4WKFD | 50-59 years, male | 30.7 |
| GTEX-13VXU-2726-SM-5LU4N | 50-59 years, male | 30.4 |
| GTEX-17F9Y-1026-SM-7IGO4 | 70-79 years, female | 30.3 |
| GTEX-1I1GS-1426-SM-COH3W | 60-69 years, male | 30.2 |
| GTEX-1KANB-1026-SM-D3L9F | 50-59 years, male | 30.2 |
| GTEX-1GN73-1026-SM-9KNV9 | 60-69 years, male | 30.1 |
| GTEX-1OJC4-0626-SM-DTXEY | 50-59 years, female | 30.1 |
| GTEX-1JMQJ-2126-SM-CNPPA | 50-59 years, male | 29.9 |
| GTEX-14JIY-1326-SM-6AJB3 | 60-69 years, male | 29.8 |
| GTEX-1JKYN-2126-SM-ARU8W | 30-39 years, male | 29.8 |
| GTEX-12WSE-0826-SM-5S2VL | 20-29 years, male | 29.6 |
| GTEX-1I1CD-0926-SM-CL55I | 60-69 years, female | 29.6 |
| GTEX-1PPGY-1826-SM-EXOJ8 | 60-69 years, male | 29.6 |
| GTEX-1F6RS-0826-SM-7P8T9 | 60-69 years, female | 29.5 |
| GTEX-111CU-0326-SM-5GZXO | 50-59 years, male | 29.4 |
| GTEX-13CF3-0426-SM-5IJEU | 60-69 years, female | 29.4 |
| GTEX-T8EM-0326-SM-3DB7F | 40-49 years, male | 29.4 |
| GTEX-14E7W-1326-SM-5RQIV | 70-79 years, male | 29.3 |
| GTEX-1JN6P-0726-SM-ARZN7 | 60-69 years, male | 29.3 |
| GTEX-1GZ4I-0726-SM-9QEHL | 50-59 years, male | 29.2 |
| GTEX-Q2AG-1026-SM-33HBW | 40-49 years, female | 29.1 |
| GTEX-ZYW4-1526-SM-5SIBA | 60-69 years, male | 29.1 |
| GTEX-145LS-1226-SM-5Q5D9 | 60-69 years, female | 28.9 |
| GTEX-POYW-1226-SM-2XCEP | 60-69 years, male | 28.9 |
| GTEX-T6MN-0826-SM-32PM4 | 50-59 years, male | 28.9 |
| GTEX-1GMRU-1426-SM-9WPPL | 50-59 years, male | 28.9 |
| GTEX-144GM-0126-SM-5Q5AX | 20-29 years, male | 28.8 |
| GTEX-13FLV-0426-SM-5KLZA | 50-59 years, male | 28.7 |
| GTEX-13FTZ-0526-SM-5IJCW | 60-69 years, male | 28.7 |
| GTEX-1GZHY-0826-SM-9OSXT | 50-59 years, female | 28.7 |
| GTEX-1AX9I-0826-SM-73KUT | 60-69 years, male | 28.5 |
| GTEX-RWSA-1126-SM-2XCAZ | 40-49 years, male | 28.5 |
| GTEX-UPK5-1126-SM-3GAEJ | 40-49 years, male | 28.5 |
| GTEX-12WSD-0826-SM-5GCNE | 60-69 years, female | 28.4 |
| GTEX-18A66-0926-SM-718BG | 60-69 years, male | 28.4 |
| GTEX-Y9LG-0526-SM-4VBRY | 30-39 years, male | 28.3 |
| GTEX-1M4P7-0826-SM-E9TJR | 60-69 years, male | 28.3 |
| GTEX-ZYY3-0926-SM-5E454 | 60-69 years, female | 28.2 |
| GTEX-1IKK5-1926-SM-A9G2V | 60-69 years, male | 28.2 |
| GTEX-1LG7Y-0826-SM-E9TJX | 50-59 years, male | 28.1 |
| GTEX-1LGRB-0226-SM-CNPQK | 50-59 years, female | 28 |
| GTEX-11NV4-1126-SM-5HL6J | 60-69 years, male | 27.8 |
| GTEX-13FTY-0126-SM-5J2NZ | 40-49 years, female | 27.8 |
| GTEX-N7MT-0126-SM-2D7VT | 60-69 years, female | 27.6 |
| GTEX-VUSG-0926-SM-3GIK6 | 50-59 years, male | 27.6 |
| GTEX-1BAJH-1326-SM-7RHFS | 70-79 years, male | 27.6 |
| GTEX-1GMR3-0226-SM-7RHIB | 40-49 years, male | 27.5 |
| GTEX-1IKJJ-0926-SM-A9G2L | 60-69 years, male | 27.5 |
| GTEX-1K9T9-0926-SM-D3L9C | 50-59 years, female | 27.5 |
| GTEX-1QP29-1226-SM-E6CQ1 | 50-59 years, male | 27.5 |
| GTEX-1H23P-1026-SM-9QEHQ | 60-69 years, male | 27.4 |
| GTEX-1QMI2-0926-SM-DTX7S | 60-69 years, male | 27.4 |
| GTEX-17EVP-0726-SM-7EWDX | 40-49 years, male | 27.3 |
| GTEX-ZVZQ-1526-SM-5N9G6 | 60-69 years, female | 27.3 |
| GTEX-XMD2-1026-SM-4WWE8 | 60-69 years, female | 27.1 |
| GTEX-13S7M-2126-SM-5S2QR | 60-69 years, female | 27 |
| GTEX-1I1GQ-1126-SM-CNNQT | 50-59 years, male | 27 |
| GTEX-13QBU-0726-SM-5J2OA | 40-49 years, female | 26.9 |
| GTEX-12KS4-0726-SM-5FQSX | 30-39 years, male | 26.8 |
| GTEX-RU72-0526-SM-2TF5Z | 50-59 years, female | 26.5 |
| GTEX-1S5VW-0926-SM-EWRNU | 60-69 years, male | 26.5 |
| GTEX-WYVS-0526-SM-3NM9W | 40-49 years, female | 26.4 |
| GTEX-11PRG-0926-SM-5EGI8 | 50-59 years, male | 26.3 |
| GTEX-1B996-0726-SM-7IGPI | 60-69 years, male | 26.2 |
| GTEX-13FHP-0726-SM-5K7YI | 50-59 years, male | 26.1 |
| GTEX-1HBPH-1326-SM-9WYSN | 40-49 years, female | 26.1 |
| GTEX-11WQC-0626-SM-5EQMF | 60-69 years, male | 26 |
| GTEX-15D1Q-0526-SM-6AJAY | 40-49 years, male | 26 |
| GTEX-OXRN-0526-SM-2I5EN | 50-59 years, male | 26 |
| GTEX-1HT8W-1426-SM-CE6S2 | 30-39 years, male | 25.9 |
| GTEX-OHPM-0526-SM-2YUMJ | 50-59 years, male | 25.8 |
| GTEX-144GO-0226-SM-5LUB1 | 60-69 years, male | 25.7 |
| GTEX-1GMR8-1426-SM-7RHHL | 50-59 years, female | 25.6 |
| GTEX-14PJM-0926-SM-6AJ9Y | 50-59 years, female | 25.5 |
| GTEX-PSDG-1126-SM-2S1ON | 50-59 years, male | 25.5 |
| GTEX-1H3VY-1426-SM-9WPOB | 50-59 years, male | 25.5 |
| GTEX-WZTO-0426-SM-3NM99 | 40-49 years, male | 25.4 |
| GTEX-VJWN-1326-SM-EVR2Z | 50-59 years, female | 25.4 |
| GTEX-1128S-0726-SM-5N9D6 | 60-69 years, female | 25.3 |
| GTEX-XOT4-1426-SM-4B65T | 60-69 years, female | 25.2 |
| GTEX-1AYCT-0726-SM-7IGNG | 50-59 years, male | 25.1 |
| GTEX-ZQG8-0326-SM-51MSE | 60-69 years, female | 24.8 |
| GTEX-1IDJI-0626-SM-C1YQD | 50-59 years, female | 24.6 |
| GTEX-ZUA1-1026-SM-4YCEA | 40-49 years, male | 24.5 |
| GTEX-15SHW-0926-SM-6LPIO | 60-69 years, male | 24.4 |
| GTEX-18A7A-1026-SM-7LT8B | 50-59 years, female | 24.4 |
| GTEX-1H3O1-0326-SM-9WPO8 | 60-69 years, male | 24.4 |
| GTEX-1RAZQ-1026-SM-EAZ4R | 30-39 years, male | 24.4 |
| GTEX-1IL2V-0726-SM-ARZMB | 70-79 years, male | 24.3 |
| GTEX-145MO-1326-SM-5Q5EF | 60-69 years, male | 24.2 |
| GTEX-1EX96-0226-SM-7RHHR | 50-59 years, male | 24.2 |
| GTEX-11EMC-0126-SM-5EGKV | 60-69 years, female | 24.1 |
| GTEX-13NYB-0626-SM-5MR47 | 40-49 years, male | 24 |
| GTEX-XV7Q-0426-SM-4BRVN | 40-49 years, female | 23.9 |
| GTEX-14PQA-1126-SM-7KUM4 | 50-59 years, female | 23.8 |
| GTEX-15EOM-5010-SM-7P8PA | 20-29 years, female | 23.8 |
| GTEX-1I6K6-0926-SM-AHZ2N | 30-39 years, male | 23.8 |
| GTEX-14BIL-1226-SM-79OME | 50-59 years, male | 23.7 |
| GTEX-18A67-1126-SM-7KFSB | 50-59 years, male | 23.6 |
| GTEX-18D9A-0226-SM-7KFSJ | 30-39 years, female | 23.6 |
| GTEX-1HB9E-0726-SM-AHZ4L | 50-59 years, male | 23.6 |
| GTEX-XBEC-1026-SM-4QASM | 50-59 years, male | 23.5 |
| GTEX-ZT9W-0726-SM-9JGG2 | 50-59 years, male | 23.5 |
| GTEX-U8XE-1426-SM-3DB8Q | 30-39 years, male | 23.3 |
| GTEX-1AX8Z-5010-SM-AHZ33 | 60-69 years, male | 23.3 |
| GTEX-1JJE9-0926-SM-CNNP9 | 70-79 years, male | 23.3 |
| GTEX-ZF28-0726-SM-4WKFU | 60-69 years, male | 23.2 |
| GTEX-11OF3-1126-SM-5986C | 60-69 years, male | 23 |
| GTEX-XXEK-0626-SM-4BRWE | 50-59 years, male | 23 |
| GTEX-1HSMO-0926-SM-A96S7 | 60-69 years, male | 23 |
| GTEX-Y5V5-0826-SM-4VBQD | 60-69 years, female | 22.9 |
| GTEX-ZLV1-0426-SM-4WWC2 | 60-69 years, female | 22.9 |
| GTEX-SJXC-1026-SM-EYYVA | 60-69 years, male | 22.8 |
| GTEX-11ZTS-1226-SM-5EQMQ | 60-69 years, female | 22.7 |
| GTEX-1I1GR-0726-SM-B2LWL | 60-69 years, male | 22.6 |
| GTEX-1JMOU-0526-SM-CNNPE | 60-69 years, female | 22.5 |
| GTEX-147GR-1226-SM-5TDCL | 60-69 years, male | 22.4 |
| GTEX-14C5O-1126-SM-5TDEH | 60-69 years, male | 22.4 |
| GTEX-1AX9K-0826-SM-731F9 | 50-59 years, male | 22.4 |
| GTEX-1NSGN-1626-SM-EXOJF | 50-59 years, male | 22.4 |
| GTEX-11GSP-0726-SM-5986L | 60-69 years, female | 22.1 |
| GTEX-18D9U-0526-SM-72D7C | 40-49 years, male | 22.1 |
| GTEX-1212Z-1026-SM-5EGJ8 | 60-69 years, male | 22 |
| GTEX-PLZ4-0726-SM-2TC6Q | 50-59 years, female | 22 |
| GTEX-SE5C-0526-SM-2XCE1 | 40-49 years, female | 22 |
| GTEX-111YS-0626-SM-5GZXV | 60-69 years, male | 21.9 |
| GTEX-11UD2-0726-SM-5EQ69 | 50-59 years, male | 21.9 |
| GTEX-11ZVC-0226-SM-731E8 | 50-59 years, female | 21.9 |
| GTEX-13N1W-0726-SM-5MR57 | 70-79 years, male | 21.9 |
| GTEX-QEG4-0526-SM-48TZD | 30-39 years, male | 21.9 |
| GTEX-ZEX8-0526-SM-DO115 | 50-59 years, male | 21.9 |
| GTEX-1B8SF-0826-SM-73KW8 | 60-69 years, male | 21.8 |
| GTEX-U3ZN-0626-SM-3DB7U | 30-39 years, female | 21.8 |
| GTEX-U8T8-2226-SM-3DB95 | 60-69 years, male | 21.8 |
| GTEX-13QJ3-1026-SM-5QGQU | 50-59 years, male | 21.7 |
| GTEX-X4XY-1026-SM-46MVX | 60-69 years, male | 21.7 |
| GTEX-15FZZ-0326-SM-6M48O | 30-39 years, female | 21.6 |
| GTEX-13X6K-1626-SM-7EWCX | 60-69 years, female | 21.5 |
| GTEX-QDT8-0926-SM-32PL2 | 30-39 years, female | 21.5 |
| GTEX-WVJS-0826-SM-4MVNR | 50-59 years, male | 21.5 |
| GTEX-14A6H-0526-SM-5NQAZ | 30-39 years, male | 21.4 |
| GTEX-14E6D-1026-SM-5S2RS | 50-59 years, male | 21.4 |
| GTEX-R55C-0526-SM-3GIKA | 40-49 years, male | 21.4 |
| GTEX-ZT9X-0326-SM-51MTE | 40-49 years, male | 21.4 |
| GTEX-1E1VI-0926-SM-7P8T3 | 50-59 years, male | 21.4 |
| GTEX-1RLM8-0626-SM-E76OW | 50-59 years, male | 21.4 |
| GTEX-TML8-0326-SM-4GICN | 40-49 years, female | 21.3 |
| GTEX-12WSG-5004-SM-7EPG9 | 50-59 years, female | 21.2 |
| GTEX-131XW-1126-SM-5EGK4 | 50-59 years, female | 21.2 |
| GTEX-13SLW-1226-SM-5S2Q7 | 70-79 years, male | 21.2 |
| GTEX-QV44-0926-SM-2S1RH | 50-59 years, male | 21.2 |
| GTEX-W5X1-0526-SM-3GILH | 40-49 years, female | 21.2 |
| GTEX-1B933-0926-SM-9OSVH | 50-59 years, female | 21.2 |
| GTEX-1IL2U-0926-SM-CKZOB | 60-69 years, male | 21.2 |
| GTEX-1J1OQ-0926-SM-CJI34 | 40-49 years, female | 21.2 |
| GTEX-146FQ-0926-SM-5LUAV | 30-39 years, male | 21.1 |
| GTEX-1GPI7-1526-SM-9JGG8 | 20-29 years, male | 21.1 |
| GTEX-12WSN-5004-SM-793CE | 40-49 years, male | 21 |
| GTEX-RM2N-0426-SM-2TF4T | 50-59 years, male | 21 |
| GTEX-1B932-0726-SM-731EY | 40-49 years, female | 20.9 |
| GTEX-14E6C-1426-SM-5ZZWH | 40-49 years, male | 20.8 |
| GTEX-16XZZ-0926-SM-7DHLI | 60-69 years, male | 20.8 |
| GTEX-RTLS-0926-SM-2TF5X | 60-69 years, female | 20.8 |
| GTEX-X585-1026-SM-46MW6 | 50-59 years, male | 20.8 |
| GTEX-1JK1U-1126-SM-CY8HK | 60-69 years, male | 20.8 |
| GTEX-QMR6-1926-SM-32PL9 | 50-59 years, male | 20.7 |
| GTEX-X4EP-0526-SM-3P5YW | 60-69 years, female | 20.7 |
| GTEX-1J8QM-0526-SM-AHZ3Y | 60-69 years, female | 20.7 |
| GTEX-11EI6-0826-SM-5985V | 60-69 years, male | 20.6 |
| GTEX-TSE9-0726-SM-3DB8C | 60-69 years, female | 20.6 |
| GTEX-1KD5A-1126-SM-CXZK6 | 50-59 years, male | 20.6 |
| GTEX-1NV5F-0926-SM-DTX8N | 60-69 years, female | 20.6 |
| GTEX-147JS-1226-SM-5RQK4 | 60-69 years, male | 20.5 |
| GTEX-SNOS-0426-SM-32PMH | 40-49 years, male | 20.5 |
| GTEX-1IDJU-0226-SM-CKZOF | 60-69 years, female | 20.5 |
| GTEX-1C6VS-1226-SM-79OO2 | 60-69 years, male | 20.4 |
| GTEX-R3RS-1026-SM-3GADF | 40-49 years, female | 20.4 |
| GTEX-ZXG5-0826-SM-5GID6 | 60-69 years, male | 20.4 |
| GTEX-1GL5R-0526-SM-9WPOQ | 50-59 years, male | 20.4 |
| GTEX-1JMPZ-1126-SM-ARU8X | 30-39 years, male | 20.4 |
| GTEX-1122O-0126-SM-5GICA | 60-69 years, female | 20.2 |
| GTEX-1HFI6-0926-SM-9WPPV | 40-49 years, male | 20.2 |
| GTEX-1KXAM-0426-SM-DHXKG | 60-69 years, male | 20.1 |
| GTEX-1LC47-1126-SM-D4P43 | 60-69 years, male | 20.1 |
| GTEX-1GF9V-1026-SM-9KNW3 | 60-69 years, male | 20 |
| GTEX-O5YT-0526-SM-32PK8 | 20-29 years, male | 19.9 |
| GTEX-QXCU-0626-SM-2TC69 | 50-59 years, male | 19.9 |
| GTEX-WRHU-0226-SM-3MJFV | 50-59 years, female | 19.9 |
| GTEX-ZAB4-0626-SM-5CVN3 | 40-49 years, male | 19.9 |
| GTEX-1F75B-1026-SM-9JGFS | 50-59 years, male | 19.9 |
| GTEX-18465-0626-SM-7LT8X | 50-59 years, male | 19.8 |
| GTEX-PLZ6-0426-SM-5IJDW | 30-39 years, male | 19.8 |
| GTEX-11DZ1-0426-SM-5H11A | 50-59 years, male | 19.7 |
| GTEX-11TUW-0526-SM-5LU9A | 60-69 years, male | 19.7 |
| GTEX-13FH7-1726-SM-5IJE7 | 50-59 years, female | 19.7 |
| GTEX-1EN7A-0826-SM-7MKFV | 40-49 years, male | 19.7 |
| GTEX-VJYA-0326-SM-3GAEX | 60-69 years, male | 19.7 |
| GTEX-ZG7Y-1026-SM-4WWDG | 50-59 years, male | 19.7 |
| GTEX-16AAH-0426-SM-7DUFM | 50-59 years, male | 19.6 |
| GTEX-1B97I-0226-SM-73KVO | 40-49 years, male | 19.6 |
| GTEX-ZA64-0326-SM-5HL8T | 20-29 years, male | 19.6 |
| GTEX-11DXZ-0726-SM-5N9C4 | 50-59 years, male | 19.4 |
| GTEX-12WSA-1026-SM-5EGHN | 60-69 years, male | 19.4 |
| GTEX-S7SE-0926-SM-2XCD6 | 50-59 years, male | 19.4 |
| GTEX-1HBPM-0826-SM-9WYSP | 60-69 years, female | 19.3 |
| GTEX-RN64-1226-SM-2TC6E | 50-59 years, male | 19.2 |
| GTEX-1HCU7-0926-SM-A96TK | 50-59 years, male | 19.2 |
| GTEX-145LT-0326-SM-5LUAD | 40-49 years, male | 19.1 |
| GTEX-16MT8-1026-SM-6LPK1 | 60-69 years, female | 19.1 |
| GTEX-17HHE-0626-SM-7DHL6 | 50-59 years, male | 19.1 |
| GTEX-1K2DU-0526-SM-D4P2Y | 20-29 years, male | 19.1 |
| GTEX-1RAZS-2226-SM-E8VMU | 50-59 years, male | 19.1 |
| GTEX-ZF3C-0926-SM-DO92P | 50-59 years, female | 19.1 |
| GTEX-1AYD5-1226-SM-7EWEP | 50-59 years, male | 19 |
| GTEX-WHPG-1426-SM-3NMBB | 50-59 years, male | 19 |
| GTEX-18D9B-1026-SM-CNPO5 | 60-69 years, male | 19 |
| GTEX-1MJK2-1126-SM-E9TJ7 | 50-59 years, male | 19 |
| GTEX-1R9PM-0426-SM-EVR3P | 20-29 years, male | 19 |
| GTEX-13O3Q-0526-SM-5KM18 | 50-59 years, male | 18.9 |
| GTEX-15RJ7-0626-SM-6M47V | 40-49 years, male | 18.9 |
| GTEX-17HGU-0926-SM-79OKO | 50-59 years, male | 18.9 |
| GTEX-RWS6-0226-SM-2XCA9 | 60-69 years, female | 18.9 |
| GTEX-S32W-0326-SM-2XCBI | 50-59 years, female | 18.9 |
| GTEX-12BJ1-1026-SM-5EGJA | 60-69 years, male | 18.8 |
| GTEX-12WSH-0126-SM-5GCO3 | 50-59 years, male | 18.8 |
| GTEX-12ZZY-0926-SM-5EQ6I | 60-69 years, male | 18.8 |
| GTEX-11LCK-0426-SM-5A5M8 | 30-39 years, male | 18.7 |
| GTEX-13RTJ-1126-SM-5S2UJ | 60-69 years, male | 18.7 |
| GTEX-QEG5-1126-SM-33HC2 | 20-29 years, male | 18.7 |
| GTEX-S33H-0626-SM-2XCBJ | 30-39 years, male | 18.7 |
| GTEX-P4PP-0526-SM-2HMKE | 30-39 years, female | 18.6 |
| GTEX-WFON-0426-SM-3GIL4 | 40-49 years, male | 18.6 |
| GTEX-1HCVE-0626-SM-A9SL2 | 50-59 years, male | 18.6 |
| GTEX-11TT1-1626-SM-5EQL7 | 20-29 years, male | 18.5 |
| GTEX-1269C-0926-SM-5FQSR | 60-69 years, female | 18.5 |
| GTEX-14BMU-0526-SM-73KW4 | 20-29 years, female | 18.5 |
| GTEX-P4QT-0526-SM-2I3EX | 50-59 years, female | 18.5 |
| GTEX-1F48J-0826-SM-9WPOD | 50-59 years, female | 18.5 |
| GTEX-1211K-0826-SM-5FQUP | 60-69 years, female | 18.4 |
| GTEX-14ABY-1126-SM-5Q5F8 | 50-59 years, male | 18.4 |
| GTEX-15G1A-0426-SM-6M468 | 30-39 years, male | 18.4 |
| GTEX-1JMQL-1126-SM-CKZOP | 50-59 years, female | 18.4 |
| GTEX-WH7G-0726-SM-3NMBM | 40-49 years, male | 18.3 |
| GTEX-ZZPT-1326-SM-5E43H | 50-59 years, male | 18.3 |
| GTEX-1GN1W-1026-SM-9OSW2 | 50-59 years, female | 18.3 |
| GTEX-117YX-1326-SM-5H125 | 50-59 years, male | 18.2 |
| GTEX-13113-5004-SM-79ONL | 60-69 years, female | 18.2 |
| GTEX-1P4AB-1026-SM-DTXES | 50-59 years, male | 18.2 |
| GTEX-1313W-0926-SM-5EQ56 | 50-59 years, female | 18.1 |
| GTEX-13JUV-0526-SM-5K7XE | 40-49 years, female | 18.1 |
| GTEX-1RAZR-0726-SM-EV7BA | 40-49 years, male | 18.1 |
| GTEX-Y3IK-0626-SM-4WWE4 | 50-59 years, female | 18 |
| GTEX-1ICLZ-0926-SM-CKZPQ | 60-69 years, male | 18 |
| GTEX-145ME-0226-SM-5S2QN | 40-49 years, female | 17.9 |
| GTEX-145MF-0726-SM-5Q5BT | 50-59 years, male | 17.9 |
| GTEX-WY7C-0426-SM-3NB3C | 50-59 years, male | 17.9 |
| GTEX-1L5NE-0726-SM-CXZKQ | 50-59 years, male | 17.9 |
| GTEX-P44H-1126-SM-48TBU | 40-49 years, male | 17.8 |
| GTEX-13PVQ-0926-SM-5IJFD | 50-59 years, male | 17.7 |
| GTEX-17F98-0226-SM-793BV | 40-49 years, female | 17.7 |
| GTEX-12WSL-1026-SM-5CVNJ | 50-59 years, male | 17.6 |
| GTEX-14DAQ-0926-SM-793AZ | 60-69 years, female | 17.6 |
| GTEX-17HII-0926-SM-79ON7 | 50-59 years, male | 17.6 |
| GTEX-1I1GU-0526-SM-B2LX2 | 40-49 years, male | 17.6 |
| GTEX-1LVAN-1126-SM-CNNRE | 50-59 years, female | 17.6 |
| GTEX-111FC-1126-SM-5GZWU | 60-69 years, male | 17.5 |
| GTEX-13W3W-0326-SM-731DS | 60-69 years, female | 17.5 |
| GTEX-14JG6-0326-SM-6AJBT | 30-39 years, female | 17.5 |
| GTEX-15UKP-1926-SM-6LPI9 | 20-29 years, male | 17.5 |
| GTEX-QDVJ-0926-SM-2I5FU | 50-59 years, male | 17.5 |
| GTEX-QDVN-0726-SM-4B64L | 50-59 years, male | 17.5 |
| GTEX-U412-0826-SM-3DB9K | 60-69 years, male | 17.5 |
| GTEX-ZLWG-0626-SM-4WWFR | 50-59 years, female | 17.5 |
| GTEX-1F7RK-0826-SM-7SB8V | 20-29 years, female | 17.5 |
| GTEX-1GN1U-1026-SM-9WYUP | 50-59 years, female | 17.5 |
| GTEX-1JMLX-0926-SM-CNNPQ | 50-59 years, male | 17.5 |
| GTEX-1OJC3-0726-SM-DTX92 | 20-29 years, male | 17.5 |
| GTEX-Y111-1026-SM-4TT22 | 50-59 years, male | 17.4 |
| GTEX-ZF29-1026-SM-4WKGC | 60-69 years, female | 17.4 |
| GTEX-13FHO-1026-SM-5KM1Q | 60-69 years, male | 17.3 |
| GTEX-1F5PK-0526-SM-7MXUA | 50-59 years, male | 17.3 |
| GTEX-1FIGZ-0326-SM-7RHGT | 40-49 years, male | 17.3 |
| GTEX-1HSMQ-0726-SM-B2LXY | 50-59 years, male | 17.3 |
| GTEX-ZPCL-0926-SM-DNZZ8 | 60-69 years, female | 17.3 |
| GTEX-1CB4I-2126-SM-793AG | 50-59 years, male | 17.2 |
| GTEX-T6MO-0426-SM-32QOI | 40-49 years, female | 17.2 |
| GTEX-U3ZM-0426-SM-3DB73 | 40-49 years, male | 17.2 |
| GTEX-WFG8-0926-SM-3GIKJ | 20-29 years, male | 17.2 |
| GTEX-1IY9M-1126-SM-A9SLV | 60-69 years, male | 17.2 |
| GTEX-Q734-0626-SM-2I3EF | 40-49 years, female | 17.1 |
| GTEX-1RDX4-1626-SM-EVR53 | 50-59 years, female | 17.1 |
| GTEX-1H1ZS-0826-SM-A9SMD | 70-79 years, male | 17 |
| GTEX-11EQ9-0226-SM-5A5JX | 30-39 years, male | 16.9 |
| GTEX-14LLW-0726-SM-5ZZVV | 70-79 years, female | 16.9 |
| GTEX-16NGA-0226-SM-718AI | 40-49 years, female | 16.9 |
| GTEX-YF7O-0626-SM-4W21R | 50-59 years, male | 16.9 |
| GTEX-1GN2E-1526-SM-7P8TD | 60-69 years, male | 16.9 |
| GTEX-14PJ5-0226-SM-5YY99 | 60-69 years, female | 16.8 |
| GTEX-146FH-1226-SM-5NQB6 | 50-59 years, female | 16.7 |
| GTEX-15CHR-0726-SM-7EPHG | 50-59 years, male | 16.7 |
| GTEX-R55D-0926-SM-3GAEU | 50-59 years, male | 16.7 |
| GTEX-YECK-0926-SM-4W214 | 60-69 years, male | 16.7 |
| GTEX-ZTPG-0926-SM-5O99H | 20-29 years, female | 16.7 |
| GTEX-183FY-0726-SM-793C3 | 20-29 years, male | 16.6 |
| GTEX-OOBK-0526-SM-2HMJJ | 40-49 years, male | 16.6 |
| GTEX-Y3I4-0426-SM-4TT29 | 50-59 years, male | 16.6 |
| GTEX-13111-0426-SM-5DUXR | 50-59 years, male | 16.5 |
| GTEX-139T6-0426-SM-5IJEM | 50-59 years, male | 16.5 |
| GTEX-13OW8-1726-SM-5L3GO | 60-69 years, male | 16.5 |
| GTEX-P4QS-0526-SM-2I3ET | 60-69 years, male | 16.5 |
| GTEX-XPVG-1026-SM-4B64Y | 50-59 years, male | 16.5 |
| GTEX-YEC3-0226-SM-DO11B | 50-59 years, male | 16.5 |
| GTEX-14XAO-0526-SM-6AJB7 | 60-69 years, female | 16.4 |
| GTEX-1QP2A-1226-SM-EVR3I | 50-59 years, male | 16.4 |
| GTEX-1A32A-0726-SM-731D4 | 50-59 years, female | 16.3 |
| GTEX-1C64O-1226-SM-79OOC | 60-69 years, male | 16.3 |
| GTEX-WK11-0526-SM-3NB3O | 50-59 years, male | 16.3 |
| GTEX-131YS-0926-SM-5IJB9 | 60-69 years, female | 16.2 |
| GTEX-13O21-3026-SM-5J2NI | 50-59 years, male | 16.2 |
| GTEX-13U4I-1426-SM-5J2M3 | 40-49 years, female | 16.2 |
| GTEX-OIZH-0526-SM-2HMKV | 50-59 years, male | 16.2 |
| GTEX-YJ8O-1826-SM-5HL82 | 40-49 years, female | 16.2 |
| GTEX-1MGNQ-0826-SM-EVR41 | 40-49 years, male | 16.2 |
| GTEX-13O61-0726-SM-5J2MD | 60-69 years, male | 16.1 |
| GTEX-X261-1026-SM-3NMDL | 50-59 years, male | 16.1 |
| GTEX-1I6K7-1226-SM-B2LVS | 40-49 years, male | 16.1 |
| GTEX-13NZ9-0926-SM-5KM12 | 50-59 years, male | 16 |
| GTEX-13S86-0626-SM-5Q5E7 | 40-49 years, male | 16 |
| GTEX-WFG7-0526-SM-3GIKI | 20-29 years, male | 16 |
| GTEX-12ZZW-0926-SM-5LZUD | 50-59 years, male | 15.9 |
| GTEX-14DAR-0226-SM-5S2PR | 50-59 years, male | 15.9 |
| GTEX-15SHV-0326-SM-6M475 | 60-69 years, male | 15.9 |
| GTEX-O5YV-0526-SM-2I5GE | 60-69 years, female | 15.9 |
| GTEX-XQ3S-0926-SM-4BOPI | 20-29 years, male | 15.9 |
| GTEX-ZPU1-0926-SM-57WDO | 40-49 years, male | 15.9 |
| GTEX-1GTWX-0626-SM-9WYU1 | 60-69 years, male | 15.9 |
| GTEX-11NUK-0826-SM-5HL4U | 50-59 years, male | 15.8 |
| GTEX-15EU6-1226-SM-6AJBE | 50-59 years, male | 15.8 |
| GTEX-17GQL-0726-SM-731BL | 60-69 years, male | 15.8 |
| GTEX-1CAMQ-1026-SM-7EPIC | 40-49 years, male | 15.8 |
| GTEX-OXRP-0526-SM-2I3EW | 60-69 years, female | 15.8 |
| GTEX-REY6-0426-SM-2TF5G | 60-69 years, male | 15.8 |
| GTEX-T2IS-0526-SM-32QP9 | 20-29 years, female | 15.8 |
| GTEX-12WSI-0826-SM-5EGKD | 50-59 years, male | 15.7 |
| GTEX-13D11-0326-SM-5LZXX | 50-59 years, female | 15.7 |
| GTEX-14PJO-0926-SM-686YT | 60-69 years, male | 15.7 |
| GTEX-1GF9X-0326-SM-7P8R9 | 40-49 years, female | 15.7 |
| GTEX-NFK9-1026-SM-2HMK1 | 40-49 years, male | 15.6 |
| GTEX-OOBJ-0526-SM-48TDK | 60-69 years, male | 15.6 |
| GTEX-QESD-0626-SM-2I5G4 | 20-29 years, male | 15.6 |
| GTEX-ZYFG-0226-SM-5GIDT | 60-69 years, female | 15.6 |
| GTEX-1I19N-0526-SM-A9G27 | 70-79 years, male | 15.6 |
| GTEX-1399S-1726-SM-5L3DI | 30-39 years, female | 15.4 |
| GTEX-15RIE-0326-SM-6PAMC | 60-69 years, male | 15.4 |
| GTEX-1CB4F-0926-SM-7DHMJ | 60-69 years, male | 15.4 |
| GTEX-TMMY-0926-SM-4TT1Z | 40-49 years, female | 15.3 |
| GTEX-U3ZH-0526-SM-3DB75 | 30-39 years, male | 15.3 |
| GTEX-1I1GV-0926-SM-B2LXM | 60-69 years, male | 15.3 |
| GTEX-1MCC2-0726-SM-EV7AL | 30-39 years, female | 15.3 |
| GTEX-S3LF-1126-SM-EVR33 | 70-79 years, male | 15.3 |
| GTEX-13NYS-1626-SM-5J2MU | 60-69 years, male | 15.2 |
| GTEX-13QJC-0526-SM-5RQKB | 60-69 years, female | 15.2 |
| GTEX-1497J-0326-SM-5Q5CN | 60-69 years, male | 15.2 |
| GTEX-14PK6-0326-SM-6AJ9S | 70-79 years, female | 15.2 |
| GTEX-17EVQ-1526-SM-79ONG | 70-79 years, male | 15.2 |
| GTEX-OHPL-0526-SM-3NM8U | 60-69 years, female | 15.2 |
| GTEX-WFJO-0326-SM-3GIL3 | 30-39 years, male | 15.2 |
| GTEX-11ZTT-0626-SM-5EQLM | 60-69 years, female | 15.1 |
| GTEX-OIZG-0526-SM-2HMLF | 50-59 years, male | 15.1 |
| GTEX-X4XX-1026-SM-4QARO | 60-69 years, male | 15.1 |
| GTEX-169BO-0226-SM-79OL1 | 50-59 years, male | 15 |
| GTEX-P78B-0926-SM-2I5FA | 40-49 years, female | 15 |
| GTEX-R55G-0826-SM-2TC5U | 40-49 years, female | 15 |
| GTEX-RU1J-0126-SM-2TF6Y | 20-29 years, female | 15 |
| GTEX-UJHI-0726-SM-3DB92 | 50-59 years, female | 15 |
| GTEX-ZY6K-0326-SM-5SIBB | 50-59 years, male | 15 |
| GTEX-11P81-0226-SM-5HL5M | 30-39 years, female | 14.9 |
| GTEX-147F3-0726-SM-5NQ9U | 50-59 years, female | 14.9 |
| GTEX-14JG1-0926-SM-5YY8W | 40-49 years, male | 14.9 |
| GTEX-O5YW-0526-SM-2YUMX | 50-59 years, male | 14.9 |
| GTEX-14C39-0326-SM-5TDDX | 40-49 years, male | 14.8 |
| GTEX-145MH-0626-SM-5NQAK | 50-59 years, male | 14.7 |
| GTEX-14AS3-0926-SM-5TDD6 | 40-49 years, female | 14.7 |
| GTEX-17KNJ-0926-SM-7IGP2 | 50-59 years, male | 14.7 |
| GTEX-1KAFJ-0326-SM-DHXJG | 50-59 years, male | 14.7 |
| GTEX-1MJIX-0626-SM-DTX8V | 40-49 years, male | 14.7 |
| GTEX-11NSD-0326-SM-5A5LS | 20-29 years, male | 14.6 |
| GTEX-132QS-0726-SM-5IJE9 | 60-69 years, male | 14.6 |
| GTEX-1B8KZ-0526-SM-73KW2 | 50-59 years, male | 14.6 |
| GTEX-QEL4-0826-SM-3GAF2 | 50-59 years, male | 14.6 |
| GTEX-1J1R8-0226-SM-AHZ3C | 40-49 years, male | 14.6 |
| GTEX-131XE-0726-SM-5HL9K | 50-59 years, male | 14.5 |
| GTEX-133LE-0526-SM-5N9EJ | 20-29 years, female | 14.5 |
| GTEX-UJMC-0726-SM-3GADX | 50-59 years, male | 14.5 |
| GTEX-Y8LW-0326-SM-4VBQ9 | 50-59 years, female | 14.5 |
| GTEX-ZF2S-0626-SM-4WKH2 | 50-59 years, female | 14.5 |
| GTEX-1H2FU-1226-SM-9KNVY | 60-69 years, female | 14.5 |
| GTEX-13YAN-1026-SM-5O9CF | 60-69 years, male | 14.4 |
| GTEX-ZYVF-1726-SM-5E443 | 50-59 years, female | 14.4 |
| GTEX-1HSMP-1326-SM-A96TR | 70-79 years, male | 14.4 |
| GTEX-13N2G-0826-SM-5IJE6 | 60-69 years, male | 14.3 |
| GTEX-OXRK-0926-SM-2HMKP | 50-59 years, female | 14.3 |
| GTEX-1J8EW-1026-SM-A96T7 | 60-69 years, female | 14.2 |
| GTEX-X15G-0626-SM-EVR32 | 50-59 years, female | 14.2 |
| GTEX-POMQ-0526-SM-3GADD | 20-29 years, female | 14.1 |
| GTEX-13O3P-1026-SM-5N9E7 | 50-59 years, male | 14 |
| GTEX-OIZI-1026-SM-3NB1K | 40-49 years, male | 14 |
| GTEX-ZE7O-0826-SM-57WCP | 50-59 years, female | 14 |
| GTEX-12WSJ-0226-SM-5GCP7 | 40-49 years, female | 13.9 |
| GTEX-1477Z-0626-SM-5NQB7 | 60-69 years, male | 13.9 |
| GTEX-12WSK-0826-SM-5CVNP | 40-49 years, female | 13.8 |
| GTEX-PW2O-0526-SM-2I3DX | 20-29 years, male | 13.8 |
| GTEX-16MTA-1226-SM-7KULL | 50-59 years, male | 13.7 |
| GTEX-X3Y1-0626-SM-3P5YS | 40-49 years, male | 13.7 |
| GTEX-ZDTT-0926-SM-5J2MS | 60-69 years, male | 13.7 |
| GTEX-13VXT-1426-SM-5LU4B | 20-29 years, female | 13.6 |
| GTEX-144GN-0426-SM-5O9AP | 50-59 years, male | 13.6 |
| GTEX-178AV-0326-SM-6LPJF | 40-49 years, male | 13.6 |
| GTEX-SUCS-0626-SM-32PM5 | 70-79 years, male | 13.5 |
| GTEX-WWYW-0926-SM-3NB2Z | 50-59 years, female | 13.5 |
| GTEX-WYJK-0826-SM-3NM8Y | 50-59 years, male | 13.4 |
| GTEX-1I4MK-0326-SM-B2LWP | 50-59 years, female | 13.4 |
| GTEX-14LZ3-0726-SM-5YYAB | 60-69 years, female | 13.3 |
| GTEX-148VI-0226-SM-5RQKA | 60-69 years, female | 13.2 |
| GTEX-15SKB-0826-SM-7KUFB | 60-69 years, male | 13.2 |
| GTEX-1A3MV-0526-SM-72D5A | 30-39 years, male | 13.2 |
| GTEX-XQ8I-1126-SM-4BOO2 | 50-59 years, male | 13.2 |
| GTEX-ZTTD-1126-SM-51MRP | 50-59 years, male | 13.2 |
| GTEX-11DXX-0626-SM-5Q5AG | 60-69 years, female | 13.1 |
| GTEX-14PJ4-0626-SM-6AJBS | 60-69 years, male | 13.1 |
| GTEX-ZAB5-0626-SM-5PNVB | 50-59 years, male | 13.1 |
| GTEX-1H11D-0726-SM-9OSWA | 40-49 years, male | 13.1 |
| GTEX-1QCLY-1026-SM-EWRNI | 30-39 years, male | 13.1 |
| GTEX-13OW5-0726-SM-5KLZK | 60-69 years, male | 13 |
| GTEX-1C4CL-0826-SM-7EWEZ | 30-39 years, male | 13 |
| GTEX-QCQG-0326-SM-2I3ES | 50-59 years, female | 13 |
| GTEX-XGQ4-0826-SM-4AT4T | 50-59 years, male | 13 |
| GTEX-1CAV2-0626-SM-7P8QE | 40-49 years, female | 13 |
| GTEX-1PPH7-0226-SM-E76Q1 | 40-49 years, male | 12.9 |
| GTEX-131XF-1026-SM-5BC6A | 60-69 years, male | 12.8 |
| GTEX-RUSQ-0626-SM-2TF5V | 50-59 years, male | 12.8 |
| GTEX-ZYT6-0526-SM-5GIEA | 30-39 years, male | 12.8 |
| GTEX-14PHY-0526-SM-664NM | 60-69 years, female | 12.7 |
| GTEX-15RJE-1026-SM-6M46X | 40-49 years, male | 12.7 |
| GTEX-15SB6-0426-SM-6LPJ5 | 50-59 years, female | 12.6 |
| GTEX-WOFM-0126-SM-3MJFE | 20-29 years, male | 12.6 |
| GTEX-PLZ5-0726-SM-2I5F9 | 50-59 years, male | 12.5 |
| GTEX-QMRM-0826-SM-3NB33 | 50-59 years, male | 12.5 |
| GTEX-13NZ8-0326-SM-5L3DF | 60-69 years, female | 12.4 |
| GTEX-ZC5H-0926-SM-5CVMZ | 40-49 years, female | 12.4 |
| GTEX-1S82P-0826-SM-EVR4P | 60-69 years, female | 12.4 |
| GTEX-13PL7-1726-SM-5J2NX | 60-69 years, female | 12.3 |
| GTEX-Y5V6-0226-SM-4V6G7 | 60-69 years, male | 12.3 |
| GTEX-15CHC-0226-SM-5YYBB | 60-69 years, female | 12.2 |
| GTEX-WHSB-0326-SM-5FQSD | 50-59 years, male | 12.2 |
| GTEX-ZZPU-0526-SM-5E44U | 50-59 years, female | 12.2 |
| GTEX-14E1K-0226-SM-62LDT | 50-59 years, male | 12.1 |
| GTEX-14E6E-0426-SM-73KUE | 30-39 years, male | 12.1 |
| GTEX-P4PQ-0526-SM-2HMKR | 60-69 years, male | 12.1 |
| GTEX-ZDYS-0426-SM-5IJEQ | 50-59 years, male | 12.1 |
| GTEX-1GMR2-0126-SM-9JGHC | 50-59 years, male | 12.1 |
| GTEX-QVUS-2026-SM-EYYW9 | 60-69 years, female | 12.1 |
| GTEX-1GZ4H-0426-SM-7MXV5 | 50-59 years, female | 12 |
| GTEX-T5JC-0826-SM-32PMC | 20-29 years, male | 11.7 |
| GTEX-Y8E4-0526-SM-4V6GC | 40-49 years, male | 11.6 |
| GTEX-1QAET-0426-SM-EXUSM | 50-59 years, male | 11.6 |
| GTEX-OIZF-0526-SM-7MXVM | 60-69 years, male | 11.5 |
| GTEX-1AX9J-1626-SM-73KUI | 60-69 years, male | 11.4 |
| GTEX-YFCO-0426-SM-4W1Z7 | 40-49 years, male | 11.4 |
| GTEX-1ICLY-0926-SM-CM2RJ | 50-59 years, male | 11.4 |
| GTEX-18QFQ-0926-SM-7LG4V | 30-39 years, male | 11.3 |
| GTEX-YEC4-0526-SM-4W21U | 40-49 years, male | 11.2 |
| GTEX-13FTX-0326-SM-5J2NG | 20-29 years, female | 11.1 |
| GTEX-1HCUA-0226-SM-A8N88 | 50-59 years, female | 11.1 |
| GTEX-PX3G-0526-SM-2I3EM | 20-29 years, female | 11 |
| GTEX-S341-0326-SM-2XCAU | 40-49 years, female | 11 |
| GTEX-Y5LM-0726-SM-4VBRP | 40-49 years, female | 11 |
| GTEX-ZPIC-0626-SM-57WDY | 40-49 years, female | 11 |
| GTEX-1JMQI-1126-SM-ARZN2 | 50-59 years, male | 11 |
| GTEX-XBED-0826-SM-47JYC | 60-69 years, male | 10.9 |
| GTEX-12696-1026-SM-5FQUV | 60-69 years, male | 10.6 |
| GTEX-V1D1-0826-SM-3P5ZA | 50-59 years, male | 10.6 |
| GTEX-XBEW-0226-SM-4AT6A | 40-49 years, male | 10.6 |
| GTEX-1JN76-0826-SM-C1YRK | 20-29 years, female | 10.6 |
| GTEX-1399U-0826-SM-5KM1P | 50-59 years, female | 10.5 |
| GTEX-XYKS-0526-SM-4BRW2 | 60-69 years, female | 10.5 |
| GTEX-YB5E-0726-SM-4VDSH | 40-49 years, male | 10.5 |
| GTEX-OXRL-0526-SM-2I3EZ | 50-59 years, male | 10.4 |
| GTEX-11P7K-0326-SM-59871 | 30-39 years, male | 9.7 |
| GTEX-13N11-0326-SM-5LUA3 | 50-59 years, female | 9.7 |
| GTEX-Q2AH-0426-SM-2I3EP | 40-49 years, male | 9.7 |
| GTEX-1H1E6-0726-SM-9WPQ4 | 30-39 years, male | 9.7 |
| GTEX-1LSVX-0626-SM-E9U59 | 60-69 years, male | 9.7 |
| GTEX-1339X-0626-SM-5IJER | 40-49 years, male | 9.6 |
| GTEX-X4LF-0526-SM-3NMB6 | 50-59 years, male | 9.5 |
| GTEX-13OVG-0326-SM-5KM57 | 60-69 years, male | 9.2 |
| GTEX-NPJ8-0326-SM-2D7VV | 40-49 years, male | 9 |
| GTEX-17HG3-0326-SM-7IGP4 | 30-39 years, female | 8.8 |
| GTEX-11I78-0126-SM-5HL6F | 50-59 years, female | 8 |
| GTEX-145MN-0926-SM-5NQBT | 30-39 years, male | 8 |
| GTEX-13NZA-1426-SM-5KM4Y | 60-69 years, male | 7.6 |
| GTEX-X5EB-0426-SM-46MVY | 40-49 years, male | 5.2 |
