# Supplementary material for: FAS gene expression, prognostic significance and molecular interactions in lung cancer
Source: Front Oncol. 2024 Oct 2;14:1473515. doi: 10.3389/fonc.2024.1473515 (PMC11479862; doi:10.3389/fonc.2024.1473515)
Supplement: Supplementary file 4 [file Table4.docx]

Supplementary Table 4: Expression of *FAS* gene on lung cancer and normal lung cell lines

| **Cell line** | ***GAPDH* CT** | ***FAS* CT** | **ΔCT** | **ΔΔCT** | **Relative Expression** |
| --- | --- | --- | --- | --- | --- |
| H1299 | 16.47504 | 18.2053 | 1.73026 | 1.85218 | 0.276974 |
| H1299 | 15.92432 | 18.07202 | 2.1477 | 2.26962 | 0.207385 |
| H1299 | 15.94839 | 18.72714 | 2.77875 | 2.90067 | 0.133909 |
| H1993 | 16.24216 | 17.7051 | 1.46294 | 1.58486 | 0.333357 |
| H1993 | 16.28957 | 17.81276 | 1.52319 | 1.64511 | 0.319722 |
| H1993 | 15.90921 | 17.1376 | 1.22839 | 1.35031 | 0.392208 |
| A549 | 14.72351 | 19.62892 | 4.90541 | 5.02733 | 0.030664 |
| A549 | 16.32681 | 20.13488 | 3.80807 | 3.92999 | 0.065608 |
| A549 | 16.43222 | 19.27282 | 2.8406 | 2.96252 | 0.12829 |
| HBE | 16.17887 | 16.10851 | -0.07036 | 0.05156 | 0.964892 |
| HBE | 16.151 | 15.90732 | -0.24368 | -0.12176 | 1.088061 |
| HBE | 15.95857 | 15.90685 | -0.05172 | 0.0702 | 0.952506 |
